# Comparative whole-genome transcriptome analysis in renal cell populations reveals high tissue specificity of MAPK/ERK targets in embryonic kidney

**DOI:** 10.1186/s12915-022-01309-z

**Published:** 2022-05-13

**Authors:** Kristen Kurtzeborn, Hyuk Nam Kwon, Vladislav Iaroshenko, Imrul Faisal, Martin Ambrož, Xing Jin, Talha Qureshi, Jussi Kupari, Anneliis Ihermann-Hella, Juho Väänänen, Henna Tyynismaa, Iva Boušová, Sunghyouk Park, Satu Kuure

**Affiliations:** 1 Helsinki Institute of Life Science, University of Helsinki, Helsinki, Finland; 2 Stem Cells and Metabolism Research Program, Faculty of Medicine, University of Helsinki, Helsinki, Finland; 3 Present address: Molecular and Integrative Biosciences Research Programme, Faculty of Biological and Environmental Sciences, University of Helsinki, Helsinki, Finland; 4 Department of Biochemical Sciences, Faculty of Pharmacy in Hradec Králové, Charles University, Hradec Králové, Czech Republic; 5 Natural Product Research Institute, College of Pharmacy, Seoul National University, 1 Gwanak-ro, Gwanak-gu, Seoul, 08826 Korea; 6 Present address: Department of Medical Biochemistry and Biophysics, Division of Molecular Neurobiology, Karolinska Institutet, SE-17177 Solna, Sweden; 7 Biomedicum Functional Genomics Unit, Helsinki Institute of Life Science and Applied Tumors Genomics Research Program, Faculty of Medicine, University of Helsinki, Helsinki, Finland; 8 Neuroscience Center, Helsinki Institute of Life Science, University of Helsinki, Helsinki, Finland; 9 Department of Medical and Clinical Genetics, University of Helsinki, Helsinki, Finland; 10 GM-unit, Laboratory Animal Center, Helsinki Institute of Life Science, University of Helsinki, Helsinki, Finland

## Abstract

**Background:**

MAPK/ERK signaling is a well-known mediator of extracellular stimuli controlling intracellular responses to growth factors and mechanical cues. The critical requirement of MAPK/ERK signaling for embryonic stem cell maintenance is demonstrated, but specific functions in progenitor regulation during embryonic development, and in particular kidney development remain largely unexplored. We previously demonstrated MAPK/ERK signaling as a key regulator of kidney growth through branching morphogenesis and normal nephrogenesis where it also regulates progenitor expansion. Here, we performed RNA sequencing-based whole-genome expression analysis to identify transcriptional MAPK/ERK targets in two distinct renal populations: the ureteric bud epithelium and the nephron progenitors.

**Results:**

Our analysis revealed a large number (5053) of differentially expressed genes (DEGs) in nephron progenitors and significantly less (1004) in ureteric bud epithelium, reflecting likely heterogenicity of cell types. The data analysis identified high tissue-specificity, as only a fraction (362) of MAPK/ERK targets are shared between the two tissues. Tissue-specific MAPK/ERK targets participate in the regulation of mitochondrial energy metabolism in nephron progenitors, which fail to maintain normal mitochondria numbers in the MAPK/ERK-deficient tissue. In the ureteric bud epithelium, a dramatic decline in progenitor-specific gene expression was detected with a simultaneous increase in differentiation-associated genes, which was not observed in nephron progenitors. Our experiments in the genetic model of MAPK/ERK deficiency provide evidence that MAPK/ERK signaling in the ureteric bud maintains epithelial cells in an undifferentiated state. Interestingly, the transcriptional targets shared between the two tissues studied are over-represented by histone genes, suggesting that MAPK/ERK signaling regulates cell cycle progression and stem cell maintenance through chromosome condensation and nucleosome assembly.

**Conclusions:**

Using tissue-specific MAPK/ERK inactivation and RNA sequencing in combination with experimentation in embryonic kidneys, we demonstrate here that MAPK/ERK signaling maintains ureteric bud tip cells, suggesting a regulatory role in collecting duct progenitors. We additionally deliver new mechanistic information on how MAPK/ERK signaling regulates progenitor maintenance through its effects on chromatin accessibility and energy metabolism.

**Supplementary Information:**

The online version contains supplementary material available at 10.1186/s12915-022-01309-z.

## Background

Mitogen-activated protein kinase/extracellular signal-regulated kinase (MAPK/ERK) is best known for its function in regulating cellular proliferation and cell cycle progression [1]. The kinase cascade leading to MEK-induced phosphorylation of ERK in normal cells is typically induced by an extracellular stimulus conveyed by growth factors and other extracellular stimuli [2]. Activation of ERK kinases leads to both transcriptional regulation of target genes and phosphorylation of cytosolic substrates [3–5]. The vast majority of MAPK/ERK functions have been identified in different cancer types, while its significant roles in morphogenesis are emerging [6–9]. Our recent work suggests that MAPK/ERK signaling is also an important regulator of kidney development [10].

Kidney development is a complex process guided by the inductive interactions and mediated by inter- and intra-cellular signaling cascades. These interactions mainly take place between and among epithelial ureteric bud (UB) tips and the metanephric mesenchyme (MM) but also receiving contributions from the stroma [11–14]. In particular, receptor tyrosine kinase (RTK)-activated signaling, induced by glial cell line-derived neurotrophic factor (GDNF)/RET and fibroblast growth factors (FGFs), plays crucial roles in regulating nephrogenesis and UB branching morphogenesis, through which the embryonic kidney grows in size and achieves its typical shape [15, 16]. Each newly formed UB tip is responsible for maintaining most of the SIX2+ nephron progenitor cells within the MM population [17–19]. Simultaneously, together with stromal signals, the UB induces MM subpopulation to undergo a stepwise mesenchymal-to-epithelial transformation and in this way ensures their timely maintenance/differentiation balance [11, 20, 21].

The embryonic kidney hosts progenitor populations that are lost either during development or soon after birth [20]. Nephron progenitors represent a heterogeneous population with differences in their cell cycle length, motility, and molecular profiles [22–26], but the causes and purpose of such variation remain obscure. The UB epithelium on the other hand is molecularly and cellularly divided into UB tips and UB stalks, which have distinct transcriptional profiles and developmental programs; the tips execute branching morphogenesis by bifurcation while differentiation takes place in the stalks [27–32]. The tips of the UB contain the progenitor cells for the entire collecting duct network, which allows fluid transport and urine excretion in the functional organ [33–36].

We and others have shown that the MAPK/ERK pathway is heterogeneously activated in several tissue types in the early developing kidney [37–39], and our recent live-imaging analysis revealed the highest activity in the UB tip cells, nephron progenitors, and differentiating nephron precursors [40]. Our previous work shows that tissue-specific knockout of MAPK/ERK in either UB or MM results in severe renal hypodysplasia through distinct mechanisms [10]. The lack of MAPK/ERK activity specifically in the UB epithelium results in thin tips which fail to change growth direction and give rise to a non-complex, oversimplified UB tree due to defective cell cycle progression, and cell-cell as well as cell-to-matrix adhesions [39]. In the MM, dynamic MAPK/ERK activation is needed for the maintenance of nephron progenitor identity through its functions in regulating the integrity of the nephrogenic niche [40].

In this study, we performed two RNA sequencing (RNA-Seq) experiments to identify transcriptional MAPK/ERK targets that participate in mediating its functions in the developing kidney. With the help of tissue-specific *Mek1/2* inactivation, green fluorescent protein (GFP) expression, and fluorescence-activated cell sorting (FACS) purification, we have identified specific targets for both the ureteric bud and nephron progenitor populations of the developing kidney but also revealed that a minor fraction of targets are shared. Our experimental data demonstrate for the first time that MAPK/ERK activation maintains the UB tip cells and participates in the prevention of its premature differentiation. Furthermore, the identified targets indicate mitochondrial metabolism as one of the key mechanisms through which MAPK/ERK activity regulates the nephron progenitors.

## Results

### Identification of transcriptional MAPK/ERK targets in renal progenitor populations

**Fig. 1:**
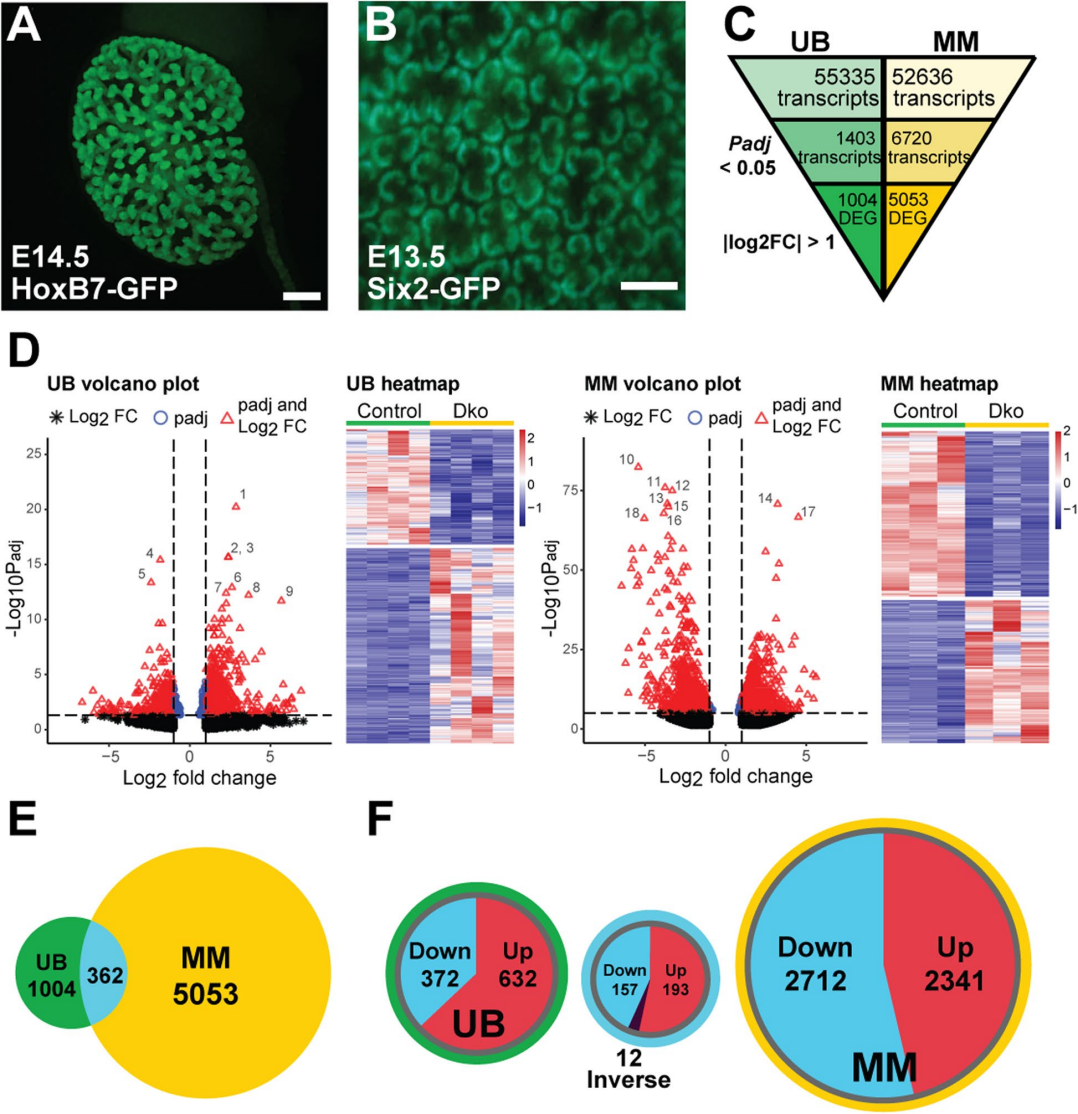
Tissue-specific bulk RNA sequencing of MAPK/ERK-deficient renal progenitor populations. **A** Whole mount image example of a mouse kidney expressing HoxB7Cre-GFP in the ureteric bud (UB) epithelium and collecting ducts at E14.5. White scale bar represents 300 μm. **B** Example whole mount image of the cortical surface of a mouse kidney expressing Six2-TGC-GFP in the nephron progenitor/metanephric mesenchyme (MM) population at E13.5. White scale bar represents 150 um. **C** RNA-Seq was separately performed on E12.5 control (*n* = 4 kidneys) and MAPK/ERK-deficient UB (*HoxB7Cre-GFP;Mek1*^*fl/fl/*^*;Mek2*^*−/−*^, *n* = 4 kidneys) and E13.5 control (*n* = 3 kidneys) and MAPK/ERK-deficient nephron progenitors (*Six2-TGC-GFP; Mek1*^*fl/fl/*^*;Mek2*^*−/−*^; *n* = 3 kidneys). Among the total reads of 55,335 and 52,636 in UB and MM, respectively, 1403 genes in the UB and 6720 in the MM were identified to be significantly differentially expressed with a cutoff of *P*_adj_ < 0.05. Further filtering with │log2fold change│≥ 1 revealed 1004 differentially expressed genes (DEGs) for UB and 5053 for MM. **D** Volcano plot and heatmap for DEGs between wildtype and dko populations in the UB (left). Similar to the UB, the volcano plot and heatmap for MM (right) demonstrate clear segregation of the control samples from those lacking MAPK/ERK (dko) activity in UB and MM populations. The most prominent DEGs are numbered in the plots and represent the following genes: 1, *Ccdc141*; 2, *Ifgbp5*; 3, *Slc40a1*; 4, *Ccnd1*; 5, *Etv5*; 6, *Atp2b4*; 7, *Samd9I*; 8, *Scn3b*; 9, *Xkr4*; 10, *Hist1h3c*; 11, *Rpl19*; 12, *Hist1h1a*; 13, *Rpl41*; 14, *mt-Rnr2*; 15, *Rpsa*; 16, *Rpl8*; 17, *Gm28439*; and 18, *Hist1h4d*. Significant DEGs in the volcano plots are marked as red dots with a statistical cutoff of *P*_adj_ < 0.05 and a magnitude threshold of │log2fold change│≥ 1. Heatmaps show downregulated genes in blue and upregulated genes in red; color intensity corresponds to the degree of differential expression. **E** Venn diagram shows 362 shared genes with differentially regulated expression in both UB and MM datasets. **F** Venn diagrams depicting up-/downregulated DEGs in UB and MM populations. Among the 1004 UB DEGs, we identified 372 genes (37%) whose expression was downregulated and 632 genes (63%) which were upregulated. Among the 362 shared DEGs between UB and MM, 157 genes (43%) were downregulated, and 193 genes (53%) were upregulated. Interestingly, 12 genes (3%) were inversely expressed, with six genes upregulated and six genes downregulated. Out of 5053 MM DEGs, 2712 genes (54%) were downregulated, and 2341 genes (46%) were upregulated

We have previously demonstrated that MAPK/ERK signaling is tissue-specifically regulating UB morphogenesis and the maintenance and differentiation of the MM [39, 40]. However, the transcriptional regulation through which MAPK/ERK functions in these tissues remains unknown. To investigate this, we utilized two different mouse models to perform RNA-Seq on UB epithelial and MM cells. To facilitate the identification of primary rather than secondary MAPK/ERK targets, the RNA was isolated from tissues at the stages where the activity of the MAPK/ERK pathway was firstly mainly depleted and morphological defects only emerging. Based on our previous characterization of the renal phenotypes in these models [39, 40], E12.5 kidneys were used for isolation of control and MAPK-deficient UB (UB_dko_; HoxB7CreGFP;Mek1^fl/fl^;Mek2^−/−^) and E13.5 kidneys for control and MAPK-deficient MM (MM_dko_; Six2-TGC^tg/+^;Mek1^fl/fl^;Mek2^−/−^) populations (Fig. 1A, B) (*n* = 2 kidneys per sample, four and three biological replicates per genotype, respectively). Principal component analysis (PCA) showed that control and *Mek1/2* double-knockout (dko) samples cluster well within their own populations but are clearly distinct from each other (Additional file 1: Fig. S1). Gene expression levels of the dko populations were normalized against the control populations, and differential expression analysis between each wildtype and UB_dko_/MM_dko_ was performed through R/Bioconductor package DESeq2 [41].

RNA-Seq identified 55,335 transcripts in the UB and 52,636 transcripts in the MM (Fig. 1C). To reveal RNAs enriched in MAPK/ERK-deficient UB and MM cells, the transcripts were filtered using a statistical cutoff of *P*_adj_ < 0.05 and a magnitude threshold of │log2foldchange│≥ 1. As a result, 1004 significantly differentially expressed genes (DEGs) were identified in the UB epithelium and 5053 in the MM (Fig. 1C, Additional file 2: Table S1 and Additional file 3: Tables S2). The high number of DEGs especially in MM probably reflects its cellular heterogeneity, which is also detected to a lesser extent in the UB [26, 30, 42–45]. Of our dataset, 63% of DEGs in the UB_dko_ are upregulated (632) and 37% downregulated (372), while DEGs in the MM_dko_ dataset are more evenly distributed and comprised 46% upregulated (2341) and 54% downregulated (2713) genes (Fig. 1D–F).

### Analysis of the MAPK/ERK transcriptomes reveals high tissue-specific fidelity

**Fig. 2:**
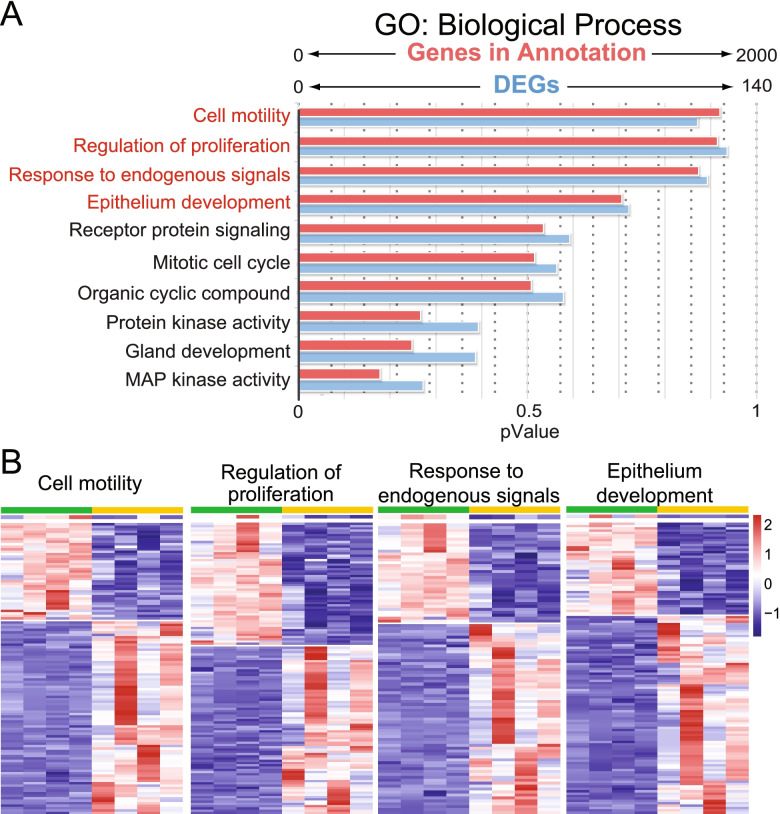
GO biological analysis of UB dataset. **A** GO biological process analysis of UB RNA-Seq data (*n* = 4 kidneys/genotype) was carried out with the ToppFun tool (application of ToppGene Suite). The figure shows the genes identified in our dataset in blue (0–140) and those within annotated functions in ToppFun in red (0–2000). **B** The heatmap analysis of the top four biological processes identified by ToppFun in the UB dataset reveals clear expressional changes, both down and up, between control (green) and MAPK/ERK-deficient UB (Dko, yellow) within the biological processes of cell motility, proliferation, response to stimulus, and epithelium development. Detailed lists of all biological processes and the genes that make them up are provided in Additional file 4: Table S3

To characterize the gene expression changes in each tissue at a general level, a Gene Ontology (GO) analysis of the DEGs of UB_dko_ and MM_dko_ cells was performed. This identified significant (*P*_adj_ < 0.05) alteration of 595 (UB) and 1035 (MM) biological processes, 80 (UB) and 257 (MM) cellular components, and 29 (UB) and 144 (MM) molecular functions, suggesting multifaceted roles for the MAPK/ERK cascade in both tissues of the developing kidney (Additional file 4: Table S3). In the UB, many of the DEGs are involved with cell motility, proliferation, response to stimuli, and epithelium development (Fig. 2). These changes are in line with, and substantiated by, the identified molecular changes and branching morphogenesis defect we previously reported for MAPK/ERK-deficient UBs [39].

**Fig. 3:**
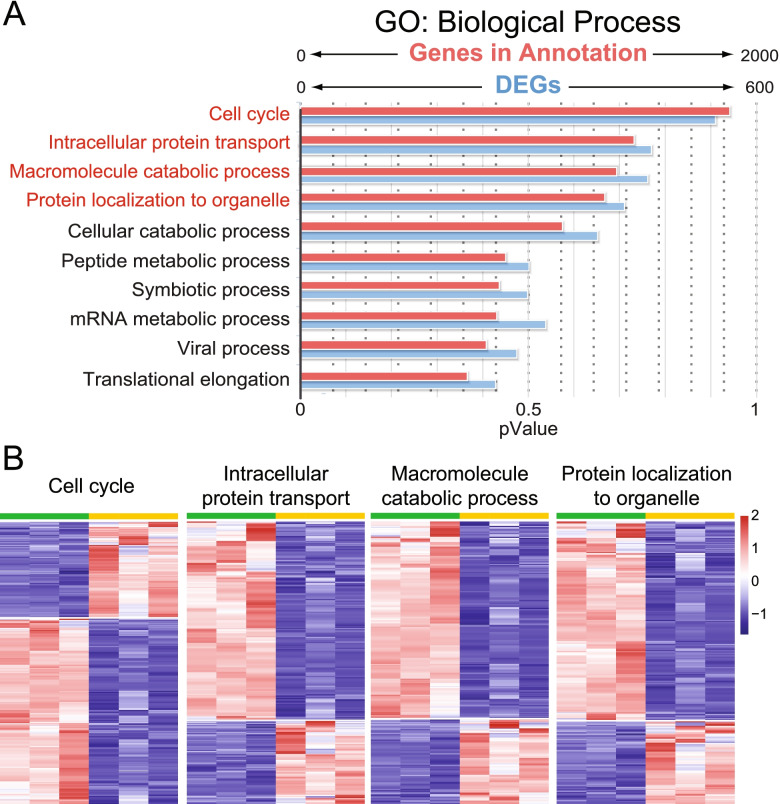
GO biological analysis on MM dataset. **A** GO biological process analysis on MM RNA-Seq data (*n* = 3 kidneys/genotype) was carried out with the ToppFun tool (application of ToppGene Suite). The figure shows the genes identified in our dataset in blue (0–600) and those within annotated functions in ToppFun in red (0–2000). **B** Heatmap analysis of the top four biological processes of MM RNA-Seq data. Genes related to cell cycle, intracellular protein transport, macromolecule catabolic processes, and protein localization to organelle have mostly opposite expression between control (green) and MAPK/ERK-deficient nephron progenitors (Dko, yellow). Detailed lists of all biological processes and the genes that make them up are provided in Additional file 4: Table S3

In the MM, the most affected biological processes relate to cell cycle, intracellular protein transport, metabolism, and protein localization (Fig. 3). GO analysis of molecular functions in both cell types revealed changes related to enzymatic activities, such as transferases and kinases but also identified distinct functions in DNA- (UB_dko_) and RNA- (MM_dko_) binding and ribonucleotide biology, including purine binding (MM_dko_) (Additional file 1: Fig. S2A-B). Cellular component analysis further confirmed the cell type-specific changes caused by MAPK/ERK deficiency and additionally revealed UB-specific transcriptional alterations that relate to organelle structures including membranes, Golgi apparatus, cell junctions, and endosomes, while in the MM, a remarkable amount of DEGs related to mitochondria, catalytic complexes, and anchoring and adherens junctions (Additional file 1: Fig. S2C-D).

Finally, we analyzed how conserved are the MAPK/ERK transcriptional targets identified by RNA-Seq in UB and MM tissues of the developing kidney. A comparison of the two datasets to one another revealed 362 shared genes (36% and 7% of DEGs in UB and MM datasets, respectively), whose expression is changed in both tissues (Fig. 1E; Additional file 5: Table S4). This suggests that the majority of the gene expression changes caused by the MAPK/ERK pathway especially in the MM of developing kidney are highly tissue-specific, as 93% of the transcriptional changes in the MM and 64% in the UB take place only in the analyzed tissue but not in the other cell type of the developing kidney.

### Shared UB and MM DEGs reflect epigenetic changes and suggest MAPK/ERK functions on histones and DNA methylation

**Fig. 4:**
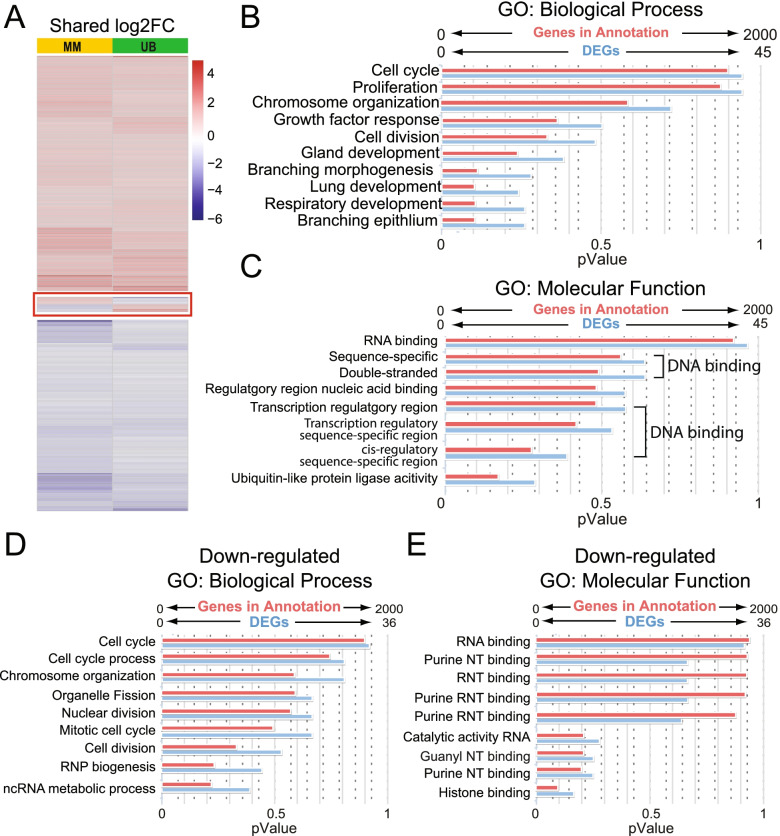
Collecting duct and nephron progenitors share a minor pool of genes whose expression is regulated by MAPK/ERK in both tissues. **A** Heatmap analysis of 362 shared genes in UB and MM. Twelve inversely expressed genes are shown in the middle of the heatmap. **B** Gene Ontology (GO) biological process analysis of shared genes between UB and MM. **C** GO molecular function analysis of shared genes between UB and MM. **D** GO biological process analysis of downregulated shared genes. **E** GO molecular function analysis of downregulated shared genes. Blue bars represent input DEGs from our RNA-Seq, and red bars represent genes in annotation. Abbreviations: ncRNA; non-coding ribonucleic acid, NT; nucleotide, RNP; ribonucleoprotein, RNT; ribonucleotide.  Detailed lists of all biological processes and the genes that make them up are provided in Additional file 6: Table S5

Detailed analysis of the genes whose expression is changed by MAPK/ERK inactivation in both UB and MM cells identified 53% upregulated transcripts (193), 43% downregulated transcripts (157), and only 12 genes (3%) which showed inverse differential expression (Figs. 1F and 4A). The inverse DEG group is composed of 11 genes with confirmed protein-coding activity (Additional file 5: Table S4). Among these, *Sprouty 1* (*Spry1*), downregulated in UB_dko_ and upregulated in MM_dko_, and *Cavin1*, downregulated in MM_dko_ and upregulated in UB_dko_, are known to inhibit MAPK/ERK signaling [46, 47] and have crucial roles in kidney development, along with *cadherin 6* and *Sox9* [48–52], also showing inverse expression in UB_dko_ and MM_dko_ (Additional file 5: Table S4).

As expected from the previously known MAPK/ERK functions [3, 53], most of the shared MAPK/ERK targets encode proteins with functions related to cell cycle, proliferation, response to growth factor, organ development, branching morphogenesis, and DNA/RNA binding (Fig. 4B, C, Additional file 6: Table S5). Among the 362 shared genes, GO analysis of the 193 upregulated DEGs revealed very few genes falling into any specific biological or molecular process category, thus demonstrating great diversity among upregulated DEGs that represent MAPK/ERK targets (Additional file 6: Table S5). On the other hand, GO analysis of the 157 downregulated shared genes reflects well with the biological processes and molecular functions identified within the total shared genes (Fig. 4D, E, compare to Fig. 4B, C). This suggests a strong and possibly universal regulatory function of MAPK/ERK cascade in processes such as cell cycle, proliferation, response to growth factor, organ development, and DNA/RNA binding.

The largest portion (13%) of the shared downregulated DEGs represent histones (Additional file 5: Table S4), and their GO analysis identified chromosome condensation and nucleosome assembly as the most affected biological processes (Additional file 6: Table S5). Indeed, 25 genes coding for histone cluster components, histone chaperones, or histone-associated kinases are downregulated in UB_dko_, and a total of 69 histone-related genes are differentially expressed in MM_dko_ (Additional file 7: Table S6). Interestingly, the most downregulated gene in each dataset is a histone cluster gene, *Hist1h2br* (H2B clustered histone 24) in UB_dko_ and *Hist1h2ai* (H2A clustered histone 13) in MM_dko_. In the MM_dko_, 57 DEGs encoding histone cluster components were downregulated, of which 28 genes showed at least a 16-fold difference and occupied a place in the top 50 downregulated genes list, suggesting a major defect. Of note, *Dnmt1*, the gene coding for the DNA (cytosine-5)-methyltransferase 1 enzyme, which is responsible for most of the DNA methylation in mouse cells, is downregulated by twofold in both MAPK/ERK-deficient UB and MM (Additional file 5: Table S4), and *Dot1l* encoding histone H3 methyltransferase is significantly downregulated in MM_dko_ cells (Additional file 3: Table S2).

Finally, we wanted to test how universally the MAPK/ERK cascade regulates the genes identified by our RNA-Seq analysis. Quantitative PCR of 20 DEGs, selected based on their high DE scores and known importance in signal transduction and/or kidney development, was first carried out in whole E12.5 kidneys cultured in control and MAPK/ERK inhibition conditions. This showed that *Ccdc69*, *Cited2*, *Cpne7*, *Dusp6*, *Etv4*, *Fjx1*, *Flrt3*, *Fosb*, *Hist1h4k*, *Hist2h3b*, *Lypla1*, *Mfng*, *Trim12a*, and genes encoding proteins related to negative regulation of ERK (*Dusp6*) and with known roles during kidney development (*Etv4*) [54–56] are significantly differentially regulated in the MAPK/ERK-deficient whole kidneys (*P* < 0.05; Additional file 1: Fig. S3A). Analysis of specific gene expressions related to chromatin regulation (*Dnmt1*, *Hist1h2br*), cell growth (*Ppan*), tip identity (*Wnt11*), cellular metabolism (*Acot7*, *Adsl*), and RTK signaling targets (*Wnt11*, *Etv4*) in isolated UB and MM populations confirmed the DEGs identified in RNA-seq (Additional file 1: Fig. S3B,C).

We also expanded our analysis of MAPK/ERK target expression to MAPK/ERK-inhibited embryonic lung and adult liver. We were able to detect that the shared transcriptional changes in DNA methyltransferase *Dnmt1*; carbohydrate derivative metabolism regulators *Acot7*, *Adsl*, and *Cad*; mitochondrial *Qtrt1*; RNA polymerase *Polr1E*; kinesin family member *Kifc1*; NME/NM23 nucleoside diphosphate kinase *Nme2*; and histone cluster gene *Hist1h2br* identified by RNA-Seq were recapitulated in the embryonic lung but not in adult liver (Additional file 8: Table S7).

### MAPK/ERK activation in the ureteric bud is required to maintain tip cell identity

Next, we focused on tissue-specific transcriptional changes caused by MAPK/ERK deficiency in the UB. It has been established that UB tips host collecting duct progenitors capable of differentiating into mature collecting duct cell types [34, 35, 54, 55]. Rutledge et al. [30] previously reported segregation between genes enriched in UB tips and stalks and identified 116 tip-enriched and 393 stalk-enriched genes in E16.5 kidneys. We compared our DEGs in MAPK/ERK-deficient UB epithelium to their published tip- and stalk-enriched lists to reveal that the UB_dko_ dataset contains 39 transcripts previously identified as tip-enriched genes and 82 transcripts identified as stalk-enriched genes (Table 1).

**Table 1:** Transcriptional changes with known tip-stalk localization identified in MAPK/ERK-deficient ureteric bud

UB-stalk-enriched genes upregulated in ***Mek1***/***2*** dko	Log2FC			UB-tip-enriched genes downregulated in ***Mek1***/***2*** dko	Log2FC
Atp6v0d2	5.82017	Paqr5	2.02776	Stmn1	− 1.0135
Thrb	5.68810	Neat1	1.99209	Rprm	− 1.0289
Vsig1	4.44785	Cpeb2	1.94871	Frem2	− 1.0412
Trim47	4.07919	Dab2	1.93896	Ctnnd2	− 1.0942
Serpinb9	3.74231	Ppp1r3c	1.90854	Uhrf1	− 1.1082
Tmem117	3.59284	Itpr2	1.88864	Stra6	− 1.1199
Wdr72	3.58091	Akr1c19	1.86199	Gfra1	− 1.1478
Trim16	3.24198	Pdlim1	1.84855	Sema6a	− 1.1662
Tspan8	3.23112	Scnn1a	1.83277	Kdm2b	− 1.1890
Igf1	3.05885	Nr3c1	1.82302	Mycn	− 1.2976
Cav1	3.04600	Igfbp7	1.72571	Slc27a6	− 1.3305
Muc20	3.02456	Elf5	1.69912	Fbln1	− 1.3432
Ccdc141	2.86843	Prlr	1.69734	Ung	− 1.3621
Tmem45b	2.86742	Scnn1b	1.68607	Dctd	− 1.3629
Gsta3	2.85412	Bmp3	1.67283	Sox8	− 1.4139
Nipal1	2.82231	Lypd6	1.63446	Nsg1	− 1.4406
Rhcg	2.80980	Slc29a1	1.63091	Cdca7	− 1.4578
Ugt2b34	2.69559	Ampd3	1.63017	Hs3st3b1	− 1.4741
Krt80	2.63243	Reps2	1.62458	Spred2	− 1.4753
Cers3	2.57988	Fam13a	1.57950	Acot7	− 1.5284
Foxa1	2.46191	Plxna2	1.57511	Fxyd6	− 1.5755
Anxa3	2.44622	Pde7b	1.53769	Fbln2	− 1.5889
Pde1a	2.41531	Dkk3	1.53697	Tmem59l	− 1.6118
Acpp	2.40222	Atp1b1	1.52502	Spry4	− 1.6210
Slc40a1	2.37983	Meg3	1.46617	Ret	− 1.7017
Slco2a1	2.36114	Foxq1	1.42632	Ror2	− 1.7602
Emp1	2.34928	AA986860	1.41052	Adamts18	− 1.8082
Fxyd4	2.22886	Parm1	1.34588	Ccnd1	− 1.8272
Pdzk1	2.19846	Slc7a8	1.31827	Cxcl14	− 1.9303
Cav2	2.14791	Itgb6	1.30563	Kank4	− 1.9339
Efemp1	2.14526	Rcan2	1.28463	Mfsd2a	− 1.9530
Shh	2.13946	Tmprss2	1.25556	Ak1	− 1.9839
Pdzk1ip1	2.09042	Sparc	1.23359	Psmc3ip	− 2.0363
Plcd1	2.08808	Krt7	1.20999	Etv4	− 2.1504
Efcab1	2.08737	Eno2	1.20676	Wnt11	− 2.1618
Aqp4	2.08663	Car12	1.19927	Psrc1	− 2.2083
Nr3c2	2.04846	Bmf	1.12381	Etv5	− 2.3936
1810019D21Rik	2.03767	Nedd4l	1.09804	Cib2	− 2.8918
Atp10b	2.03048	Cystm1	1.02906	Nrtn	− 5.3538

**Fig. 5:**
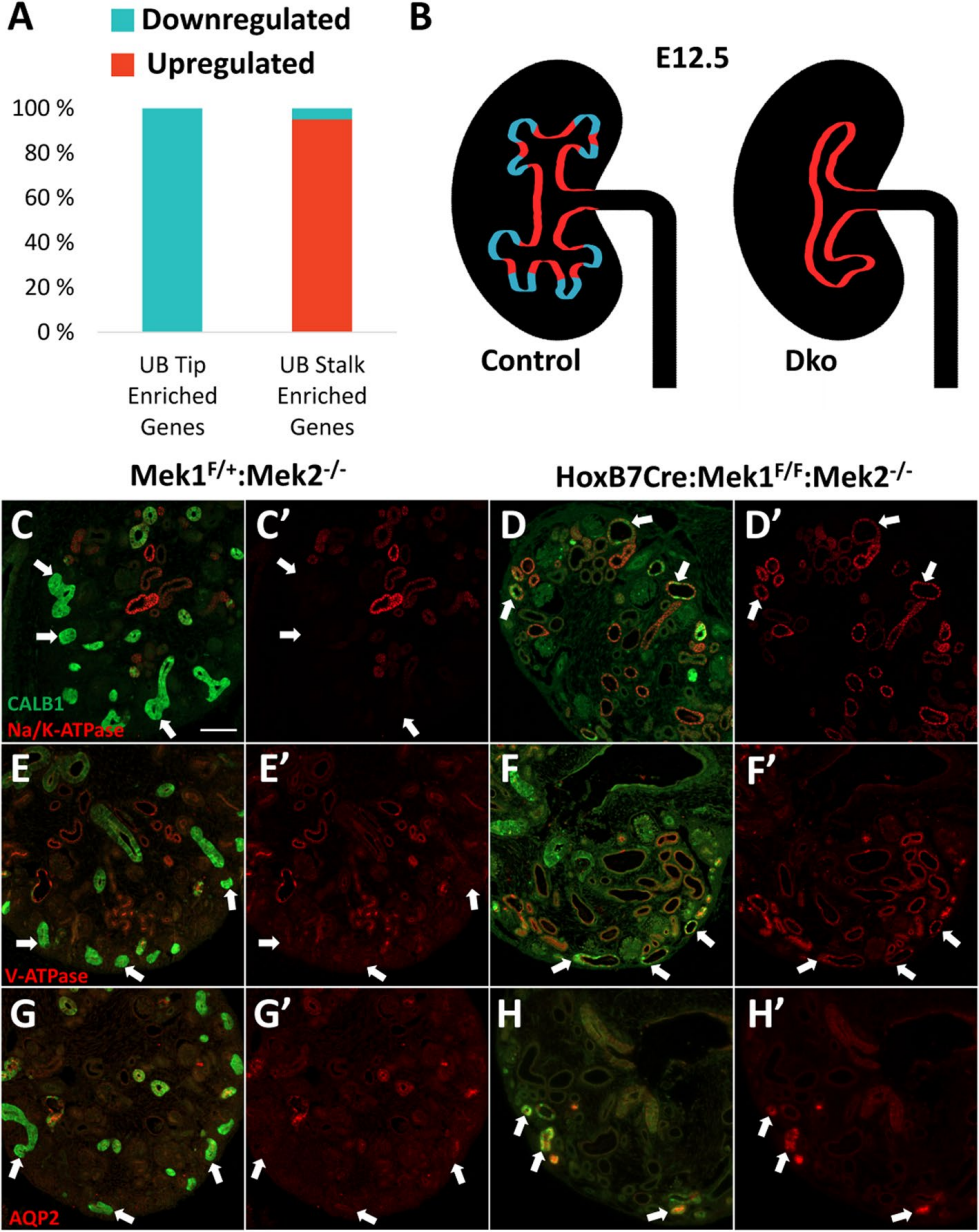
MAPK/ERK-deficient UB tips prematurely differentiate into UB stalk-like cells. **A** Comparison of differentially expressed genes in UB_dko_ to a published dataset of genes with enriched expression in either the UB tip or UB stalk (Rutledge et al.) revealed that of the previously identified UB tip-enriched genes that are differentially expressed in the UB_dko_, all (39/39 genes) are downregulated (blue). Of the UB stalk-enriched genes differentially expressed in the UB_dko_, 95% (78/82 genes) are upregulated (red). **B** Schematic representation of the gene expression changes between the UBs of wildtype (control) and *HoxB7Cre-GFP;Mek1*^*fl/fl*^*;Mek2*^−*/−*^ (dko) kidneys at E12.5. It is known that the control kidney has distinct gene expression signatures in the UB tips (blue) compared to the UB stalks (red). Additionally, the control kidney exhibits a stereotypic branching pattern. We have shown previously that in the absence of MAPK/ERK activation, the UB has a noticeable branching defect. Our analysis here revealed that MAPK/ERK-deficient UB epithelium loses the expression of tip specific genes and instead shows upregulation of stalk-enriched genes (*n* = 4 kidneys/genotype). **C–H’** Immunofluorescent staining of the E16.5 kidney paraffin sections. Calbindin-1 (CALB1) labels UB epithelium (green), and arrows indicate CALB1-positive cortical UB epithelium. **C**-**D’** Na/K-ATPase (red), a marker of principal cells in the mature collecting duct, is absent in the cortical UB tips of **C**, **C’** control kidneys but is detected in CALB1-positive cortical UB epithelium in **D**, **D’** UB_dko_. Similarly, V-ATPase-B1/2 (red), a marker of intercalated cells in the mature collecting duct, is absent in the cortical epithelium of **E**, **E’** control kidneys but localized to cortical epithelium of **F**, **F’** UB_dko_. **G**, **G’** Aquaporin-2 (AQP2), another marker of intercalated cells, is also absent from control but **H**, **H’** detected in UB_dko_ cortical epithelium. Scale bar, 100 μm

Interestingly, all (100%) the genes previously classified as tip-enriched and found in our dataset (39/39) are downregulated, including the most highly enriched tip gene *Wnt11* identified by Rutledge et al. [30] (Table 2, Fig. 5A, Additional file 1: Fig. S3B). On the contrary, almost all (95%) previously identified UB stalk-enriched genes are upregulated in MAPK/ERK-deficient UBs (78/82). At the individual gene level, 18 genes out of 39 total genes have known functions in kidney development and are downregulated in UB_dko_ including well-characterized *Etv4*, *Etv5*, *Frem2*, *Gfra1*, *Ret*, and *Spred2* with central roles in UB morphogenesis (Table 2). The upregulated stalk-enriched genes include *Aqp4*, *Cav1*, *Krt7*, *Shh*, and *Serpinb9* with previously reported involvement in kidney development [74–78]. Interestingly, such a dramatic change in the tip-stalk gene expression pattern suggests that MAPK/ERK activity functions to maintain the progenitor status of the progenitor cells within the UB tip niche.

**Table 2:** Downregulated tip-enriched genes with known functions in kidney development. 19 out of 39 genes with previously characterized tip-specific expression pattern play well-known functions in ureteric bud morphogenesis and are cited in references column

Gene	References
*Nrtn*	Davies et al. [57]
*Etv5*	Lu et al. [54]; Kuure et al. [55]
*Wnt11*	Majumdar et al. [58], O’Brien et al. [59]
*Etv4*	Lu et al. [54]; Kuure et al. [55]
*Cxcl14*	Schmidt-Ott et al. [28]
*Ccnd1*	Ihermann-Hella et al. [39]
*Adamts18*	Rutledge et al. [60]
*Ror2*	Nishita et al. [61]
*Ret*	Schuchardt et al. [62]; reviewed in Costantini [63]
*Spry4*	Zhang et al. [64]
*Hs3st3b1*	Rutledge and McMahon [65]
*Sox8*	Reginensi et al. [50]
*Mycn*	Stanton et al. [66]; reviewed in Hohenstein et al. [67]; Pan et al. [68]
*Gfra1*	Cacalano et al. [69]; Enomoto et al. [70]; reviewed in Costantini [63]
*Ctnnd2*	Rutledge et al. [30]
*Frem2*	Jadeja, et al. [71]; Kiyozumi, et al. [72]
*Rprm*	Magella et al. [73]
*Stmn1*	Li et al. [30]

To further examine the underlying gene regulatory networks involved in UB tip identity, we next utilized the web-based StemChecker [79], which compares an input gene list to those published for known transcription factor target gene sets. Using the DEGs from the UB RNA-Seq, we compared the enrichment of up- and downregulated genes among transcription factor target genes (Additional file 1: Fig. S4). Among the downregulated genes, we observed a significant overrepresentation of genes targeted by pluripotency master regulators *Oct4* and *Sox2* as well as proliferation regulators *Myc*, *Max*, and *E2f4* [80–82]. In contrast, the results for upregulated genes revealed that they were the main targets of polycomb complex components *Ezh2*, *Suz12*, and *Ring1B* [83]. These findings provide additional evidence that MAPK-deficient UB tip cells lose their progenitor state and proliferative/self-renewal capacity while increased expression of genes targeted by polycomb complexes, which function to repress developmental regulatory genes in mouse embryonic stem cells [84], further supports this.

To examine the transcriptome-suggested change in cell type fate in vivo, we analyzed the maturation status of MAPK/ERK-deficient UB epithelium at the protein level in embryonic kidneys. UB tips host collecting duct progenitors that give rise to the mature collecting duct epithelium with two main differentiated cell types, namely intercalated and principal cells [85, 86]. Immunofluorescence staining was performed with markers detecting principal (aquaporin 2 (AQP2), Na/K-ATPase) and intercalated (V-ATPaseB1/2) cells of the collecting duct (Fig. 5). In control kidneys at E16.5, AQP2 localized solely to the medullar principal cells, and V-ATPase was found in the medullar intercalated cells while, as expected, the cortical UB tips lack all three tested differentiation markers (Fig. 5C, E, G). Notably, all three tested markers, AQP2, V-ATPase, and Na/K-ATPase, were more abundantly detected in the UB_dko_ kidneys where they additionally localized to the cortical-most regions of ureteric buds representing the tip population (Fig. 5D, F, H). These results support our transcriptional findings and show that UB cells lacking MAPK/ERK activity prematurely differentiate into mature collecting duct cell types. In conclusion, MAPK/ERK activity, through maintaining the UB tip identity, is essential for preventing premature differentiation of the UB epithelium.

### MAPK/ERK activity regulates genes for mitochondrial functions in nephron progenitors

**Fig. 6:**
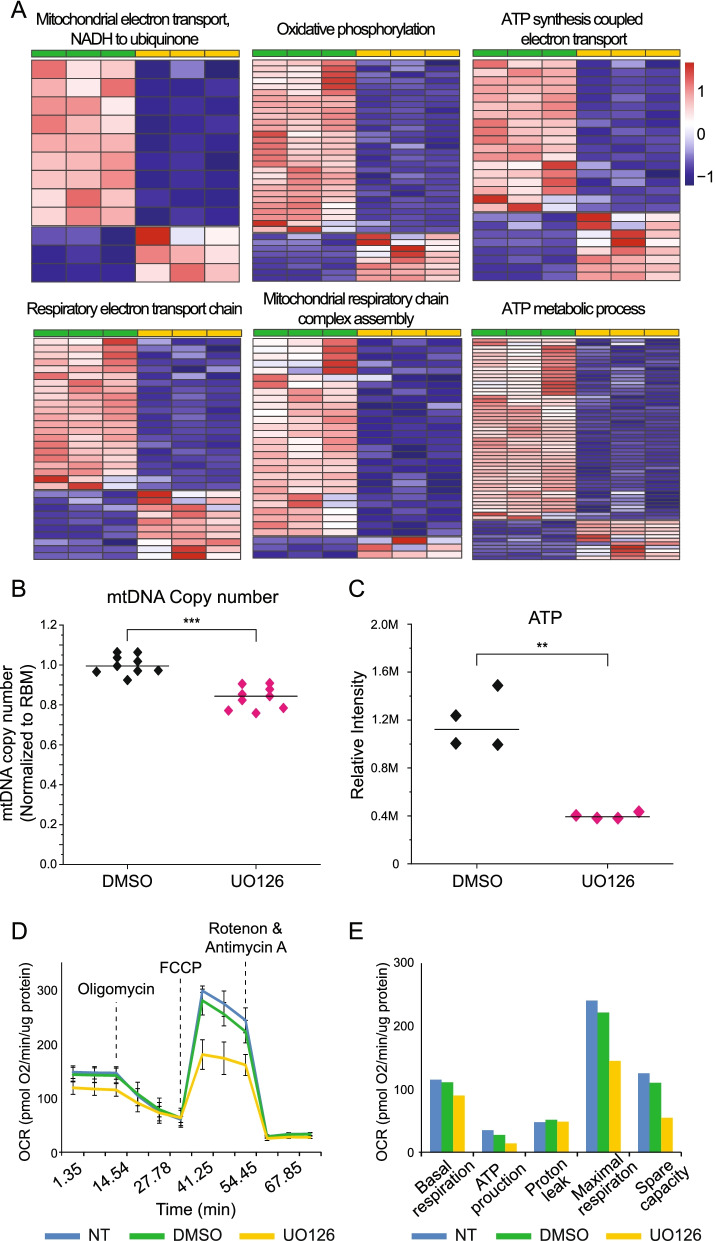
Gene expression changes in MAPK/ERK-deficient nephron progenitors suggest defects in mitochondrial functions. **A** Further heatmap analysis on the MM dataset revealed that mitochondria-related biological processes are affected in the absence of MAPK/ERK activation. The identified genes are listed in Additional file 9: Table S8. **B** Mitochondrial DNA copy number analysis was performed on control (DMSO, *n* = 9) and MEK1/2-inhibited (U0126, *n* = 9) E12.5 kidneys by real-time PCR. Mitochondrial DNA was measured by the analysis of its *12S* expression against nuclear DNA quantification by *Rbm* expression. **C** Quantification of ATP from mK4 cell line derived from embryonic kidney mesenchyme by LC/MS (*n* = 4 replicates for each DMSO and U0126). ATP concentrations are normalized against total protein concentration (Additional file 10: Table S9). **p < 5 × 10^−3^, ***p < 5 × 10^−6^. **D** Oxygen consumption rate (OCR) of mK4 cell line was measured by a Seahorse XF analyzer. For the OCR measuring, ATP synthase inhibitor (oligomycin), protonophore uncoupler (FCCP), and ETC inhibitors (rotenone and antimycin A) were added at the indicated points (*n* = 4 replicates for each DMSO and U0126). **E** Basal respiration, ATP production, proton leak, maximal respiration, and spare capacity measures are shown in different samples. Error bars represent standard deviation (S.D.). NT, non-treated mK4 cells; DMSO, DMSO-treated mK4 cells as a control; U0126, MEK inhibitor U0126-treated mK4 cells

Our GO analysis of the MM dataset revealed that several interesting cellular components related to the mitochondria are differentially expressed upon MAPK/ERK inactivation (Additional file 1: Fig. S2D, Additional file 1: Fig. S3C). Further characterization of the MM dataset with mitochondria-related biological processes identified DEGs in MM_dko_ that involve processes of mitochondrial respiratory chain function (Fig. 6A). Because most of the transcripts related to mitochondrial functions appear downregulated in the MM_dko_ dataset (representing nephron progenitors), we wanted to distinguish between the possibilities of general mitochondria count versus mitochondrial functionality being affected by MAPK/ERK activity. The analysis of mitochondrial DNA (mtDNA) copy number in control and MAPK/ERK-deficient kidneys at E12.5 revealed that, indeed, mitochondria amount per cell, as determined by mtDNA copy number against nuclear DNA copy number, was statistically significantly reduced (Fig. 6B). This suggests that the MAPK/ERK pathway is required for the mitochondrial replenishment in actively dividing nephron progenitors.

Finally, we wanted to further examine the possibility that diminished mitochondria fail to fuel cells in the absence of MAPK/ERK activation, a hypothesis also supported by the finding that adenosine triphosphate (ATP)-related metabolic activity was one of the mitochondrial functions affected by MAPK/ERK deficiency (Fig. 6A). To test the functional capacity of the mitochondria upon MAPK/ERK inhibition, we measured the actual ATP amounts in embryonic kidney cells by using liquid chromatography–mass spectrometry (LC/MS). This analytic quantification revealed a significant decrease of ATP amount in MAPK/ERK-inhibited cells (Fig. 6C). In line with this, Seahorse analysis showed decreased ATP production and further indicated impaired basal respiration, maximal respiration, and spare capacity in MAPK/ERK-inhibited cells (Fig. 6D, E). Taken together, our RNA-Seq of MM_dko_ identified a decline in mitochondria-related biological processes, which are functionally supported by lower mtDNA copy number, oxygen consumption, and quantitative ATP level. Consequently, our observations suggest that MAPK/ERK deficiency in nephron progenitors leads to mitochondrial defects, which possibly contribute to the defects in progenitor self-renewing and provide grounds for more in-depth follow-up studies.

## Discussion

Although the importance of the MAPK/ERK signaling pathway in the early developing kidney has been demonstrated by us and others [10, 27, 38, 87], the tissue-specific impacts of MAPK/ERK signals on transcriptional regulation are not fully understood. Moreover, MAPK/ERK activation in tissues of developing kidneys is highly dynamic [40], suggesting differences in cellular responses. The MAPK/ERK activation pattern has been intimately linked with the type of transcriptional response in the signaling cell, where dynamic activation typically results in more efficient transcription of target genes, which however are subject to heavy post-translational regulation [88]. In order to elucidate deep insights into the transcriptional MAPK/ERK targets at the genetic level, we here performed RNA-Seq on cells isolated from tissue-specific MAPK-deficient kidneys (UB_dko_ and MM_dko_) which are composed of heterogenous progenitors. RNA-Seq was carried out at the earliest time point where we have previously verified the loss of MAPK/ERK activity but where the kidney size is not impacted [39, 40]. In this way, our aim was to identify the primary MAPK/ERK targets in the given tissue, though this strategy may miss transcripts affected by partial downregulation of the pathway activity and also capture secondary targets. Profiling at different developmental stages might provide more information, but due to complex genetics of conditional deletion of multiple genes requiring an excessive amount of breeding mice, it would be tedious, expensive, and contrary to 3R principles. Moreover, different ERK activation modes and strengths are now known to regulate distinct transcriptional outputs resulting in varied cell fate decisions [88–90]. The next strategy to take would be single-cell RNA-Seq profiling of ERK high and low activity cells in UB and MM [40, 91]. Comparison of targets identified with the help of Förster Resonance Energy Transfer (FRET)-biosensor visualization of activation strengths to the bulk RNA-Seq targets identified here will deliver more insight not only into progenitor heterogeneity but also to their MAPK/ERK dependence.

**Fig. 7:**
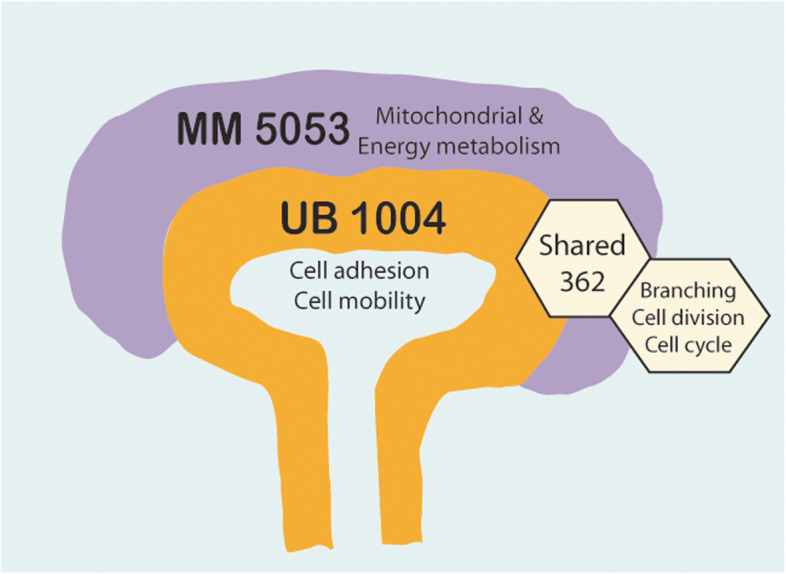
Schematic summary of MAPK/ERK functions in renal progenitor populations. With the help of RNA-Seq and tissue-specific Mek1/2 knockout, kidneys representing MAPK/ERK-deficient ureteric bud (UB) epithelium (orange) and nephron progenitor cells (purple), 1004 and 5053 differentially expressed genes were identified in MM and UB, respectively. Major changes in MM have a profound association with mitochondrial and energy metabolism. In the UB, the majority of changes correlate with cell adhesion and cell mobility functions which our further experimentation demonstrates to result in failure to maintain tip identity, resulting in the premature differentiation of the collecting duct epithelium (not shown here). Most of the changes in the 362 shared genes are related to branching, cell division, and cell cycle

Gene expression profiles of whole wildtype kidneys at different developmental stages have been reported through microarray analysis [92], but the complete lack of spatial definition of gene expression is a major limitation. Since the introduction of RNA-Seq [93], it has provided notable insights into early-stage renal development [45, 94]. Here, we provide the first tissue-specific MAPK/ERK deficiency genetic profiles for UB and MM in the early developing kidney. The results suggest that MAPK/ERK targets are highly tissue-specific regardless of the pathway’s rather universal control of proliferation through histone availability, adhesion, and energy metabolism via regulation of metabolic enzyme expression (Fig. 7). Tissue-specific transcriptional responses identified here are not unexpected as not only different growth factors but also the nature of MAPK/ERK activation, whether dynamic or sustained, induces distinct target gene expression and cell-fate outcomes [88–90, 95, 96]. As MM and UB are regulated by somewhat overlapping but hierarchically different growth factor cues [97, 98] and we have earlier demonstrated both sustained and dynamic MAPK/ERK activation in developing kidney tissues [40], target gene tissue specificity is in line with their complex expressional control.

One of the first RNA-seq results revealed rather broad transcriptional heterogeneity in a seemingly uniform nephron progenitor compartment [45], and this has since then been confirmed by others [26, 43, 44]. Brunskill and co-workers identified a few DEGs that are interesting and possibly relevant for our results. They observed some cells, mainly located in the periphery of the nephron progenitor niche, that simultaneously expressed nephron progenitor marker *Six2* and stromal marker *FoxD1* [19, 99] suggesting multilineage capacity for some early nephron progenitor cells [45]. *Six2* was not significantly differentially expressed in MAPK/ERK-deficient nephron progenitor cells. However, of the ten highest elevated gene expression in the uninduced MM compared to induced MM identified by Brunskill, six were downregulated in our MM dataset (*Ctsz*, *Osr1*, five *Coq9* family members (3, 6, 7, 8a, and 8b), *Arf2* family member *Arf5*, *Cpxm1*, and *Ccm2*). Interestingly, MAPK/ERK-deficient MM also showed increased expression of *FoxD1* and some other Fox genes possibly suggesting lineage confusion in the absence of MAPK/ERK activation.

The interactions between ERK and chromatin have been characterized in several cell and tissue types, including both human and mouse embryonic stem cells, where ERK targets histone and other transcriptional control genes [100, 101] to regulate nucleosome assembly and chromatin availability. Similar global changes as we detected in nucleosome and chromatin assembly/organization were also revealed at least in embryonic lung and placenta development [6, 102]. Moreover, sustained MEK inhibition or its genetic loss in embryonic stem cells results in epigenetic and genomic changes, which severely deteriorate the developmental potential [103]. The interactions between MAPK/ERK and chromatin are revealed here for the embryonic kidney, where 2.5% of the DEGs in UB_dko_ (25) and 1.4% of the DEGs in MM_dko_ (69) represent histones, and the largest portion (13%) of the shared downregulated DEGs fall into the chromosome condensation and nucleosome assembly category as well. DNA replication in rapidly proliferating cells requires not only histone recycling but also efficient histone transcription, which is partially ensured by their clustered localization in the genome [104, 105]. This protects newly synthesized DNA by packaging, which is required for the maintenance of chromatin integrity in the development and differentiation [106]. The amount of histones is inherently linked to DNA content [107–109], and decreased histone transcription in the absence of MAPK/ERK activation likely relates to delays in G1/S phase cell cycle progression, which we previously reported for both mutant tissues [39, 40]. MAPK/ERK is also known to directly regulate the transcriptional machinery and chromatin structure [100, 110, 111] providing an additional mechanism for the identified differential histone expressions in UB and MM.

Recently, overall chromatin landscapes were reported for embryonic and early postnatal nephron progenitors [112], but the regulation of open and closed chromatin structures in the developing kidney remains largely unknown. We found here that the DNA methylator *Dnmt1*, which is responsible for most of the DNA methylation in mouse cells, together with *Uhrf1*, which mediates DNMT1 recruitment to DNA, are significantly downregulated in both MAPK/ERK-deficient UB and MM. UHRF1 recruits DNMT1 to DNA during the S-phase of the cell cycle to ensure the maintenance of DNA methylation marks in replicating cells [113]. Downregulation of both *Uhrf1* and *Dnmt1* suggests defects in the maintenance of methylation status and thus cell identity in the kidney progenitor cells lacking MAPK/ERK activity, as previously reported for nephron progenitors lacking *Dnmt1* [114]. Such a mechanism behind the failure to maintain renal progenitors in the MAPK/ERK-deficient kidneys is further supported by a significant reduction of *Dot1l*, a histone 3 lysine 79 methyltransferase, specifically in nephron progenitors, where *Dot1l* function was recently shown indispensable for their maintenance and differentiation [115].

Our RNA-Seq results in UB_dko_ showed that in the absence of MAPK/ERK, the UB tip progenitors have decreased expression of several genes which are previously shown to be tip-specific and simultaneously upregulate many of the known stalk-specific genes [30]. Furthermore, GO biological process analysis revealed 62 genes involved in the negative regulation of cell differentiation and 25 genes involved in stem cell differentiation. Most of these genes are well characterized as regulators of progenitor state and self-renewal, notably in cell types of the developing kidney are *Sema3a* [116], *Shh* [76, 117], and *Dnmt1* [114]. Together, these suggest that MAPK/ERK activity in the UB tips is required for the maintenance of tip cells and possibly for collecting duct progenitor characteristics, as in the absence of MAPK/ERK activation, they become transcriptionally more like UB stalk cells. Indeed, our protein level analysis verified the RNA-Seq-identified transcriptional changes in the UB-specific model of MAPK/ERK deficiency and revealed premature and ectopic differentiation of principal and intercalated collecting duct cells in the UB tips. Such a requirement for MAPK/ERK activation in tip cell identity is further supported by the finding that the MAPK/ERK pathway mediates GDNF’s regulatory effect on collecting duct progenitors [36]. The loss of tip/progenitor identity also explains our previous observations that in the absence of MAPK/ERK activation, the UB tip cells fail to expand and generate new branches [39].

Transcriptional profiling of nephron progenitors identified a remarkable amount of DEGs related to proliferation and mitochondrial metabolism. Defects in mitochondrial function may cause apoptosis, and we observed decreased dko cell yields (38% in UB and 63% in MM) in FACS used for RNA-Seq. However, our earlier studies have demonstrated that cell death is not primarily affected in either of the genetic model kidneys [39, 40] supporting the deficits in energy metabolism. It has been reported that energy source influences the renewal-differentiation balance in nephron progenitors, which prematurely differentiate if glycolysis is prevented [118]. Our analysis showed that the overall mitochondria amount per cell in MAPK/ERK-deficient cells is reduced, which may perturb normal energy source balance in highly proliferative nephron progenitors due to diminished use of pyruvate for oxidative phosphorylation. Previously, p53, one of the well-known MAPK/ERK targets [90] also downregulated in our MM_dko_ dataset, was shown to regulate energy metabolism in nephron progenitors [119]. Interestingly, the nephron progenitors lacking p53 are rapidly diminished, disorganized, and show defects in cellular adhesion, thus closely resembling the phenotype we have described for MAPK/ERK-deficient nephron progenitors [40]. Taken together, the MAPK/ERK pathway likely contributes to the regulation of nephron progenitor self-renewal and maintenance through its effects on ATP availability and energy metabolism, which will be further characterized in future studies.

## Conclusions

With the help of the transcriptional MAPK/ERK target identification, we reveal here new mechanisms through which extracellularly induced guidance cues are mediated to two tissues of developing kidney, namely the branching ureteric bud epithelium and nephron progenitors. Our results show that MAPK/ERK activity in the tips of the UB protects epithelial cells from premature differentiation and thus allows continuous kidney growth through repeated tip bifurcation needed for new branching events [39]. Our RNA-Seq data suggests that the maintenance of collecting duct progenitor cell identity in the tips of the UB epithelium depends on the active MAPK/ERK cascade. We verified this at the protein level and show that in the absence of MAPK/ERK activity, the epithelial cells prematurely differentiate into collecting duct cell types, thus losing the capacity to branch and grow. This suggests that MAPK/ERK activity operates as a rather late gatekeeper of undifferentiated versus differentiated fate.

Our experimental data in the nephron progenitors demonstrate that MAPK/ERK activation maintains normal mitochondrial turnover and thus their metabolism that is required in these highly proliferative embryonic progenitors [120]. Based on the identified differences in the target gene signatures between ureteric bud and nephron progenitors, we suggest earlier function for MAPK/ERK activity in nephron progenitor cell fate decision hierarchy. This is supported by many other tissue-specific stem cells showing energy dependence similar to nephron progenitors, which together with the known functions of MAPK/ERK in the regulation of cell cycle progression, suggests mitochondrial energy regulation as a general mechanism through which MAPK/ERK pathway controls cellular turnover [89, 121, 122].

## Methods

### Mouse lines, kidney dissection, and tissue-specific cell isolation

*Six2*-TGC, *HoxB7*Cre-GFP, *Mek1*-floxed, and *Mek2*-null mice and their genotyping have been described previously [9, 18, 123, 124]. Mice are kept in mixed genetic backgrounds containing C57BL6/Rcc and 129/SvEv strains. Animal husbandry and procedures were approved by the EU legislation and the Finnish Animal Care and Use Committee. For sequencing, metanephric kidneys were dissected from E12.5 *HoxB7*Cre-GFP and E13.5 *Six2*-TGC embryos. Genotypes were analyzed by PCR. Experimental samples were tissue-specifically null for all four Mek1/2 alleles (referred to as dko, double knockout), HoxB7Cre-GFP;Mek1^fl/fl^;Mek2^−/−^ (UB_dko_) and Six2-TGC;Mek1^fl/fl^;Mek2^−/−^ (MM_dko_). Control samples for the MM were Six2-TGC;Mek1^fl/+^;Mek2^+/+^ and Six2-TGC;Mek1^fl/+^;Mek2^+/−^, and controls for the UB were HoxB7Cre-GFP;Mek1^fl/+^;Mek2^+/−^, HoxB7Cre-GFP;Mek1^+/+^;Mek2^+/−^, and HoxB7Cre-GFP;Mek1^fl/+^;Mek2^+/+^. Both kidneys from each embryo were combined, dissociated using 0.25% trypsin-EDTA with gentle pipette aspiration, and FAC-sorted based on endogenous GFP expression using a Sony SH800Z Cell Sorter.

### RNA isolation for sequencing and data analysis

RNA for sequencing was isolated from FAC-sorted cells by a standard protocol for chloroform/isopropanol extraction. Isolated RNA was treated with DNase I, following the manufacturer’s instructions (Thermo Fisher Scientific—04/2016, rev. B.00) with phenol/chloroform extraction. Total RNA quality and quantity were estimated by using the Bioanalyzer RNA Total Pico (Agilent Technologies, Inc.) and Nanodrop analysis. Examples of quality assurance are shown in Additional file 1: Fig. S5.

Library preparation was done using NuGen Ovation Solo. The NuGen kit with single-end reads is such that the library is first generated, after which ribosomal RNA is removed before amplification and sequencing with random primers. Sequencing with NextSeq was performed at BIDGEN DNA Sequencing and Genomics Laboratory (University of Helsinki). bcl2fastq2 Conversion Software was used to convert BCL files to FASTQ file format and demultiplex samples. Sequenced reads were trimmed for adaptor sequence and masked for low-complexity or low-quality sequence using Trimmomatic. Trimmed reads were mapped to GENCODE Mus musculus Release M23 reference genome GRCm38 using STAR aligner (2.6.0c). Counts per gene were calculated using featureCounts included as a part of the subread package [125] using the GENCODE Mus musculus Release M23 annotation file.

After receiving the matrix of raw gene counts, the data was normalized using DESeq2 R-package (v.3.6.2) [41], and the sample clustering was visually inspected by drawing PCA plots to detect possible sample outliers. The DESeq2 package uses a negative binomial generalized linear model to detect differentially expressed genes from the data. As part of the analysis, DESeq2 also performs independent filtering where the mean of normalized counts is used as a filter statistic. This step removes genes with insufficient expression levels and in addition samples with extreme count outliers are detected by Cook’s distance. Finally, multiple testing adjustment of *p*-values is done with the Benjamini-Hochberg procedure. From the *Six2*-TGC mice, three control and three dko samples were compared. From the *HoxB7*Cre-GFP mice, four control and four dko samples were compared. All control samples contained Cre and at least two *Mek* alleles. The results were filtered to include genes with a fold change greater than 2 (│log2FC│ ≥ 1) and false discovery rate *P*_adj_ < 0.05.

### Tissue cultures and RNA isolation for qPCR

E12.5 kidneys and lungs were cultured on filters in a Trowell-type system for 24 h in the presence of 15 μM U0126 chemical inhibitor or the same volume of DMSO vehicle control, as described previously [39]. For tissue-specific qPCR validation, UBs and MMs were manually isolated after enzymatic collagenase treatment by microdissection from E12.5 kidneys [126]. Previously reported culture mediums supplemented with 15 μM U0126 or equal amount of DMSO were used for 3-h culture of both tissues: 50 ng/ml HGF, 5 ng/ml GDNF, 25 ng/ml FGF2 for UBs [126], and 50ng/ml FGF for MM [127]. Tissues were dissociated in TRIzol Reagent (Invitrogen) assisted by aspiration through a 25-gauge needle. RNA was isolated for whole embryonic kidneys and lungs following the same procedure as above (extraction from FAC-sorted cells). Complementary DNA was generated from total RNA (1–5 μg per reaction) with the SuperScript IV first-strand synthesis system, following the manufacturer’s instructions (Invitrogen—07/2017, rev. A.0).

The livers from three adult C57BL6 mice were sliced to 5 mm diameter and 150 μm thickness. Three precision-cut slices per condition were cultured for 24 h with 15 μM U0126 or equal volume DMSO. All three precision-cut slices per condition were combined in TRIzol, and RNA isolation and reverse transcription were performed in the same manner as above.

### Quantitative-PCR

Gene-specific primers for qPCR were designed using NCBI Primer-BLAST. Primer sequences can be found in Additional file 11: Table S10. For qPCR, we used SensiFAST SYBR No-ROX kit (Bioline) with Bio-Rad CFX384 thermal cycler. The Bio-Rad CFX Manager software was used for gene expression analysis. Relative mRNA expression levels were calculated using the comparative Ct method, and individual expression values were normalized to the expression of eukaryotic translation initiation factor 3 subunit A (*Eif3a*) and STT3, subunit of the oligosaccharyltransferase complex, homolog B (S. cerevisiae) (*Stt3b*) for kidney and lung samples and to beta-actin (*Actb*) for liver samples. The results were combined from two biological replicates (kidneys, lungs, and livers originating from two separate litters or individuals), at least two technical replicates per biological replicate, and five replicates for each gene/condition combination within each run.

### Functional gene enrichment analysis

For the functional gene enrichment analysis, we applied the ToppFun application from ToppGene Suite (https://toppgene.cchmc.org), which provides functional enrichment analysis of candidate genes in many biological categories [128, 129]. ToppFun provides a set of advanced feature annotation tools for associating functional terms with gene lists through clustering algorithms. GO enrichment was performed to analyze the DEGs identified at the functional level, with a false discovery rate set to *p* < 0.05.

### Immunofluorescent staining and imaging

Kidneys were collected at the embryonic stages indicated in the text. Samples were fixed overnight in 4% paraformaldehyde (PFA), embedded in paraffin using an automated tissue processor, cut into 5-μm-thick sections, and allowed to dry overnight. Immunofluorescent staining of paraffin sections was performed following standard protocol with xylene-alcohol deparaffination series followed by heat-induced antigen retrieval in 20 mM Tris-HCl, 1 mM EDTA buffer at pH 8.5. After cooling to room temperature, tissue sections were blocked 1 h in 50 mM Tris-HCl, 100 mM NaCl, 0.1% Tween-20, at pH 7.5 (TNT) supplemented with 10% FBS, followed by incubation with primary antibodies at 4 °C overnight, TNT washing, and 1 h incubation with secondary antibodies. Primary antibodies were used as follows: AQP2 (1:300, Sigma), CALB1 (1:800, Santa Cruz), Na/K-ATPase (1:300, Hybridoma Bank), and V-ATPaseB1/2 (1:100, Santa Cruz). Detection was performed with Alexa Fluor-conjugated secondary antibodies (1:400; Jackson Immuno Research Laboratories). Additionally, fluorescent dye Hoechst (Invitrogen) was used at 1:1000 dilution. Epifluorescence imaging was performed with Zeiss Axio Imager.M2 outfitted with Hamamatsu Orca Flash 4.0 LT B&W camera and Zeiss Zen 2 software.

### Metabolite extraction from cultured cells

The mK4 cell line originally derived from embryonic kidney mesenchyme was cultured as previously described [130]. U0126 (15 μM) was used to mimic genetic MEK ablation in the cell line [39], and after 24-h treatment, cells were harvested with 0.25% Trypsin-EDTA, and cell pellets were applied to metabolite extraction for ATP quantification by double phase methanol-chloroform extraction [131]. In brief, cell pellets were re-suspended with 600 μl of methanol and chloroform (2:1 v/v ratio), and two cycles of the following steps were repeated: quick frozen with liquid nitrogen for 60s, thawing at room temperature for 2 min. After two cycles, 200 μl of chloroform and 200 μl of distilled water were added, and samples were centrifuged at 15,000*g* for 20 min at 4 °C. The upper water-soluble phase was collected and dried with a centrifugal vacuum evaporator. The protein aggregates in the middle phase were collected and re-solubilized with 6 M urea buffer for total protein concentration measurement. After speed vacuum, the dried pellets were stored at − 80 °C until analysis.

### ATP measurement by liquid chromatography-mass spectrometry (LC-MS)

Frozen cell metabolite extracts were dissolved in 200 μl solvent (water:acetonitrile = 1:1 v/v ratio) and centrifuged at 15,000*g* for 10 min at 4 °C before injection to LC-MS. Chromatographic separation was performed on a ZIC-HILIC column (3.5 μm, 200 Å, 100 × 2.1 mm; Merck Millipore) using an Acquity UPLC system (Waters, MA, USA). The column temperature was 35 °C with a flow rate of 0.4 mL/min and the auto-sampler cooler temperature was set at 4 °C with an injection volume of 2 μl. Analytes were eluted with a mobile phase composed of 10 mM ammonium acetate in water with pH 6.8 (A) and 10 mM ammonium acetate in water:acetonitrile solution (1:3) with pH 6.8 (B). Gradient conditions were as follows: 0–1 min kept B as 100%, 1–7 min declined B from 100 to 20%, 7–11 min kept B as 20%, 11.1 min set B as 100%, and kept the composition until 23 min. Mass spectrometry experiments were performed on a Q Exactive Focus Orbitrap Mass Spectrometer (Thermo Fisher Scientific) equipped with HESI sources. Data were acquired in negative mode. The measurement conditions were as follows: HESI source spray voltage of 2.5 kV, Aux gas heater temperature of 350 °C, sheath gas flow of 50, aux gas flow of 15, sweep gas flow of 2, capillary temperature of 320 °C, and S-lens RF level of 50. Scan range for the full MS experiment was 65–975 m/z with resolution as 70,000. For the PRM experiment targeting ATP measurement, the resolution was set as 35,000 and the collision energy was 20 eV. Full MS and PRM were executed simultaneously to acquire data.

### Data processing and relative quantification of ATP

The target metabolite ATP was double confirmed using the MS2 spectrum pattern compared with Metlin, an online MS database, and high-resolution m/z from the full MS experiment. Peak intensity lists of the ATP were acquired using the Xcalibur Quan Browser software (Thermo Fisher Scientific). All data were normalized by total protein concentration before comparing in groups.

### Mitochondrial DNA copy number measurement

E12.5 mouse kidneys supplemented with DMSO or 15 μM U0126 (48 h) were cultured as described above and followed by DNA isolation. The kidneys (< 5 mg) were suspended in 600 μl TRIzol Reagent (Invitrogen) and vortexed for 30 s for complete homogenization. One sample volume (600 μl) of 70% ethanol was added to enhance nucleic acid precipitation. The mixture was loaded onto the RNeasy MinElute spin column and centrifuged at 8000×*g* for 15 s. The flow-through containing DNA, proteins, and other contaminants was loaded onto a NucleoSpin® Tissue Column (MACHEREY-NAGEL). The DNA binding, washing, and drying steps were performed according to the manufacturer’s protocol (MACHEREY-NAGEL—01/2017, Rev. 17) followed by elution in 50 μl buffer BE (5 mM Tris/HCl, pH 8.5).

The set of primers (Table S10) used for mitochondrial encoded 12S ribosomal RNA (12S rRNA) and nuclear-encoded ribosomal binding protein 15 (RBM15) were used for mitochondrial DNA (mtDNA) copy number analysis. The mtDNA copy number was calculated by normalizing 12S rRNA amount against RBM15 amount.

### Seahorse analysis of mitochondrial metabolism

The cellular oxygen consumption rates (OCR) of mK4 cell line were observed using a Seahorse XF96e analyzer (Seahorse Bioscience; Agilent Technologies). The XF96e sensor cartridge activation with 200 μl of XF96 calibrant solution buffer was performed during the overnight on non-CO_2_ incubator at 37 °C. Two days before the OCR measurement, 70,000 mK4 cells/well were seeded onto the XF96 cell culture microplate and cultured in the regular culture medium (DMEM) on CO _2_ incubator at 37 °C. One day after, 15 μM of U0126 and DMSO were added, and cells were incubated for 24 h on CO _2_ incubator at 37 °C. One hour prior to the OCR measurement, the medium was changed to serum-free and bicarbonate-free DMEM medium supplemented with 10 mM Glucose, 5 mM pyruvate, and 5 mM glutamine, pH 7.4, and incubated for 1 h in a non-CO_2_ incubator at 37 °C. The Seahorse analyzer sequentially injected 1 μM of oligomycin (ATP synthase inhibitor), 1μM of FCCP (protonophore uncoupler), and 1 μM of Rotenone and Antimycin A (ETC inhibitors) and measured OCR before and after injection at indicated time points. The observed OCR was normalized against total protein amount (micrograms). To determine the mitochondrial function, basal respiration, ATP production, proton leak, maximal respiration, and spare respiration capacity were calculated according to the manufacturer’s instructions. In detail, non-mitochondrial respiration rate; minimum rate measurement after rotenone/antimycin A injection, basal respiration; (last rate measurement before first injection) − (non-mitochondrial respiration rate), maximal respiration; (maximum rate measurement after FCCP injection) − (non-mitochondrial respiration), proton leak; (minimum rate measurement after oligomycin injection) − (non-mitochondrial respiration), ATP production; (last rate measurement before oligomycin injection) − (minimum rate measurement after oligomycin injection), spare respiration capacity; (maximal respiration) − (basal respiration).

## Supplementary Information

**Additional file 1: Fig. S1.** Principal component analysis (PCA) for RNA-Seq data. **Fig. S2.** Functional gene enrichment analysis of UB and MM datasets. **Fig. S3.** Quantitative PCR analysis of MAPK/ERK targets in embryonic kidney, MM and UB tissues. **Fig. S4.** Transcription factor target-enrichment in MAPK/ERK-deficient UB. **Fig. S5.** Examples of RNA quality assurance studies performed prior proceeding to sequencing of actual samples.

**Additional file 2: Table S1.** The differentially expressed genes in UBdko dataset.

**Additional file 3: Table S2.** The differentially expressed genes in MMdko dataset.

**Additional file 4: Table S3.** Gene ontology analysis of UBdko and MMdko datasets.

**Additional file 5: Table S4.** Genes differentially expressed in both UB and MM datasets.

**Additional file 6: Table S5.** Gene ontology analysis of shared differentially expressed genes.

**Additional file 7: Table S6.** Identified histone-related genes differentially expressed in UB and MM datasets.

**Additional file 8: Table S7.** Analysis of MAPK/ERK target genes in extra-renal tissues. The gene expression changes identified in embryonic kidney were also quantified by qPCR in E12.5 mouse lungs and adult liver slices cultured for 24h in the presence of MEK inhibitor U0126. The analyzed genes are listed in “Gene” column followed by Yes/No to indicate whether the expression pattern observed in MAPK/ERK-deficient embryonic kidney was recapitulated in the extra-renal tissue. “Regulation” column shows whether the given gene expression was up- or down-regulated by MEK inhibition and indicates the potential statistical significance (*=*p*<0.05; **=*p*<0.01; ***=*p*<0.001).

**Additional file 9: Table S9**. Gene list for heatmaps in Fig. 6.

**Additional file 10: Table S9.** Raw data values for measurements of ATP, protein amount and calculations of normalized ATP intensities.

**Additional file 11: Table S10.** qRT-PCR primer sequences.

## Data Availability

The data supporting the findings of this study are available within the article and its supplementary materials that includes original data of ATP measurements. Specifically, RNA sequencing (RNA-seq) dataset that supports the findings of this study is available in the Gene Expression Omnibus repository, accession number GSE174229 (https://www.ncbi.nlm.nih.gov/geo/query/acc.cgi?&acc=GSE174229) [132].

## Abbreviations

ATP: Adenosine triphosphate
BCL: Binary base call
DEG: Differentially expressed gene
DMSO: Dimethyl sulfoxide
dko: Double-knockout
FACS: Fluorescence-activated cell sorting
FBS: Fetal bovine serum
FGF: Fibroblast growth factor
FRET: Förster (or fluorescence) resonance energy transfer
GFP: Green fluorescent protein
GDNF: Glial cell line-derived neurotrophic factor
GO: Gene Ontology
LC/MS: Liquid chromatography–mass spectrometry
MAPK/ERK: Mitogen-activated protein kinase/extracellular signal-regulated kinase
MM: Metanephric mesenchyme
MM_dko_: MAPK-deficient metanephric mesenchyme
mtDNA: Mitochondrial DNA
qPCR: Quantitative PCR
*P*_adj_: *P*-value adjusted
PCA: Principal component analysis
PCR: Polymerase chain reaction
PFA: Paraformaldehyde
RNA-seq: RNA sequencing
RTK: Receptor tyrosine kinase
UB: Ureteric bud
UB_dko_: MAPK-deficient ureteric bud
U0126: Chemical MEK inhibitor
3R: Refine, reduce, replace
