# Supplementary material for: Comparative whole-genome transcriptome analysis in renal cell populations reveals high tissue specificity of MAPK/ERK targets in embryonic kidney
Source: BMC Biol. 2022 May 13;20:112. doi: 10.1186/s12915-022-01309-z (PMC9102746; doi:10.1186/s12915-022-01309-z)
Supplement: Supplementary file 1 — Additional file 1: Fig. S1. Principal component analysis (PCA) for RNA-Seq data. Fig. S2. Functional gene enrichment analysis of UB and MM datasets. Fig. S3. Quantitative PCR analysis of MAPK/ERK targets in embryonic kidney, MM and UB tissues. Fig. S4. Transcription factor target-enrichment in MAPK/ERK-deficient UB. Fig. S5. Examples of RNA quality assurance studies performed prior proceeding to sequencing of actual samples. [file 12915_2022_1309_MOESM1_ESM.docx]

**Additional file 1: Fig. S1-4**

**Figure S1.** Principal component analysis (PCA) for RNA-Seq data

**
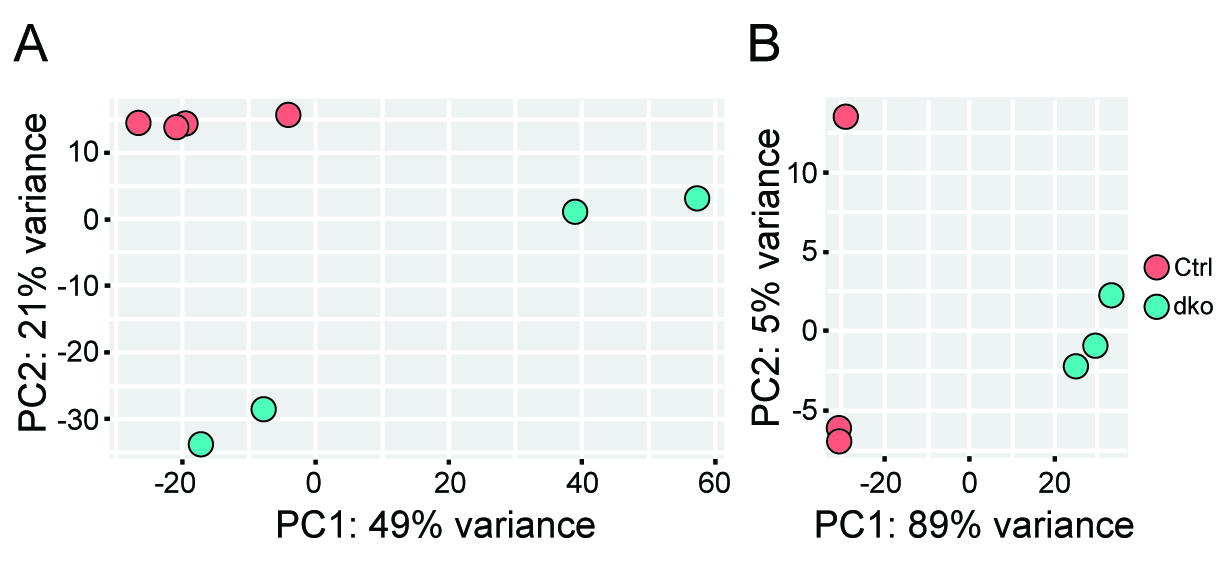
**

**(A)** PCA performed based on normalized gene counts shows that 70% of variations in the UB data (n= 4 kidneys/genotype) are explained by the first two PCs (PC1; 49% and PC2; 21%). **(B)** Similar PCA result demonstrates that 94% of variations in the MM data (n= 3 kidneys/genotype) are explained by the first two PCs (PC1; 89% and PC2; 5%). Each dot indicates an individual sample, and the same color indicates biological replicates: control (Ctrl) and dko are presented in orange and blue, respectively. Principal component 1 (PC1) and principal component 2 (PC2) were identified by Bioconductor DESeq2.

**Figure S2.** Functional gene enrichment analysis of UB and MM datasets


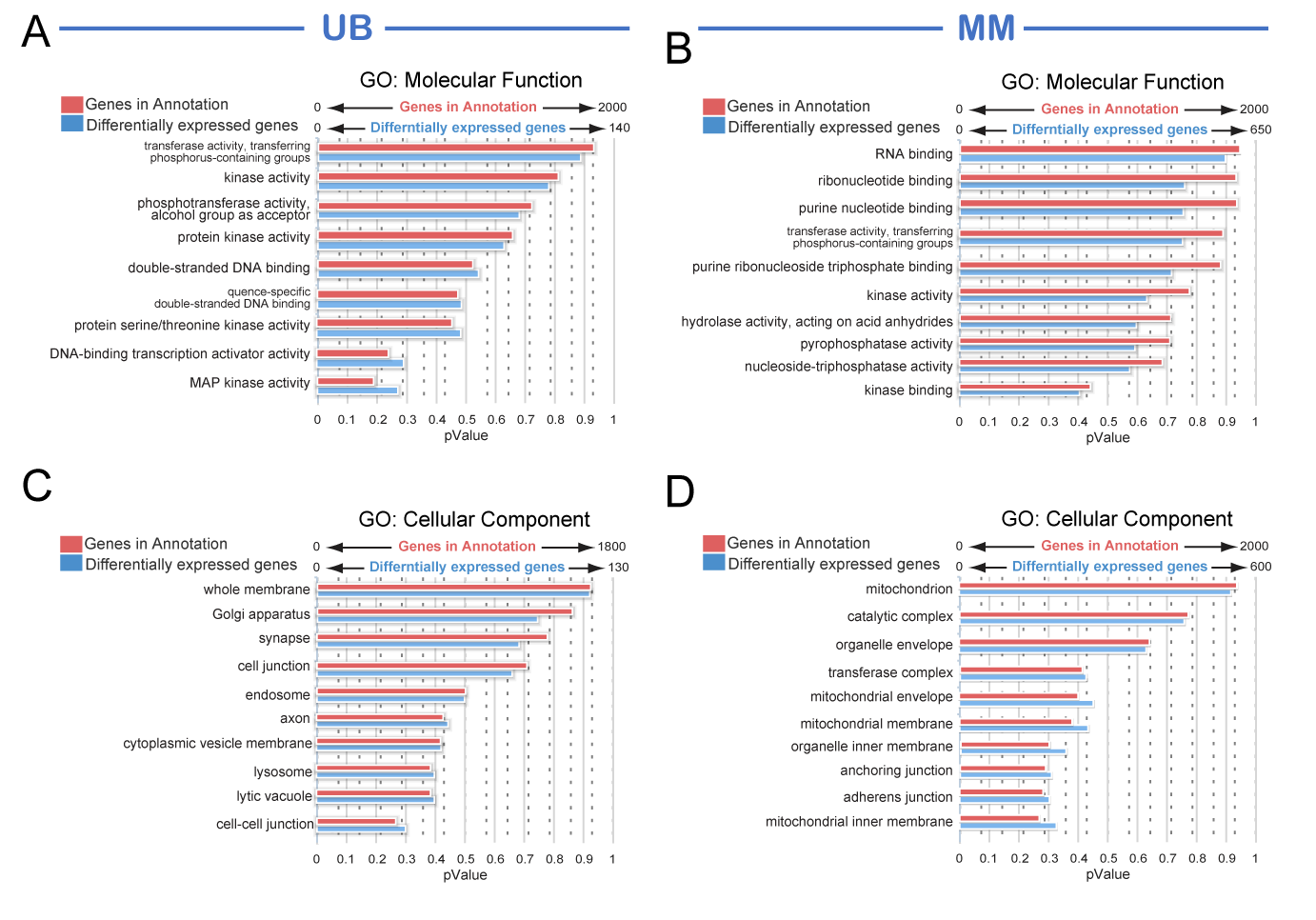


GO molecular functions of **(A)** the UB (n= 4 kidneys/genotype) and **(B)** MM (n= 3 kidneys/genotype) RNA-Seq data. GO cellular component analysis in **(C)** UB and **(D)** MM RNA-Seq data. Blue bars represent input DEGs from our RNA-Seq and red bars represent total genes in annotation.

**Figure S3**. Quantitative PCR analysis of MAPK/ERK targets in embryonic kidney, MM and UB tissues

**A**


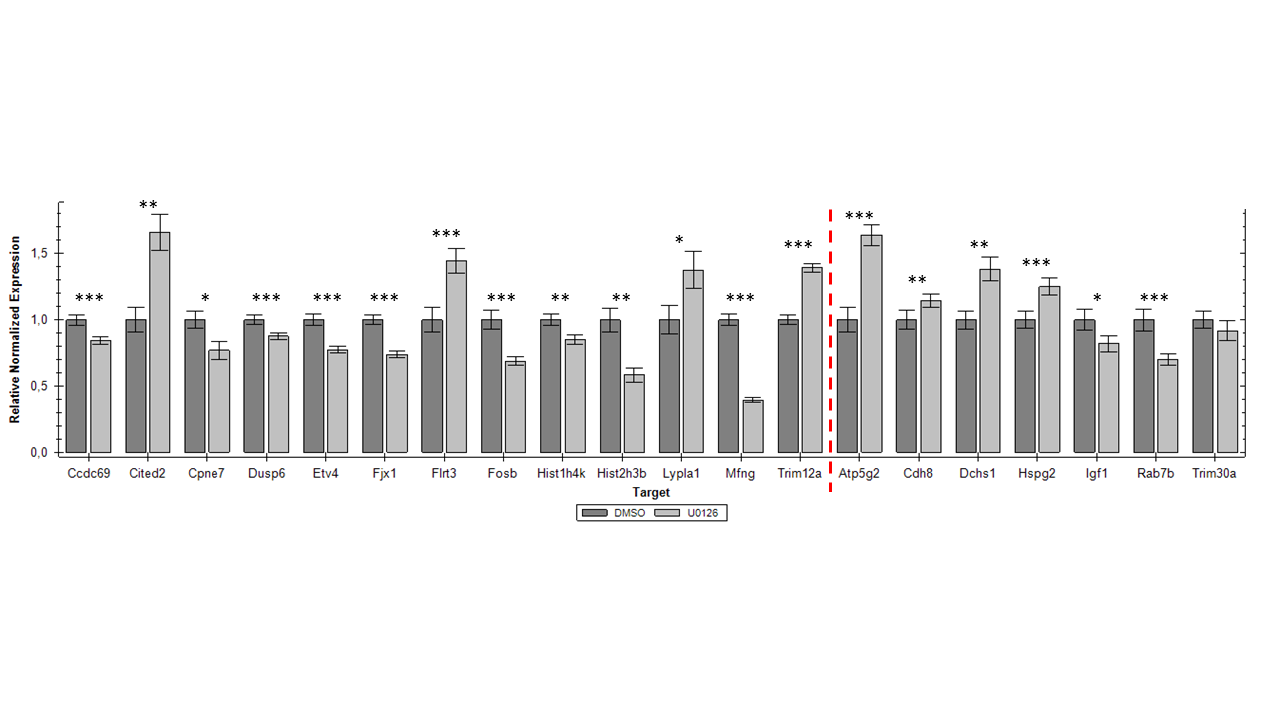


**B**


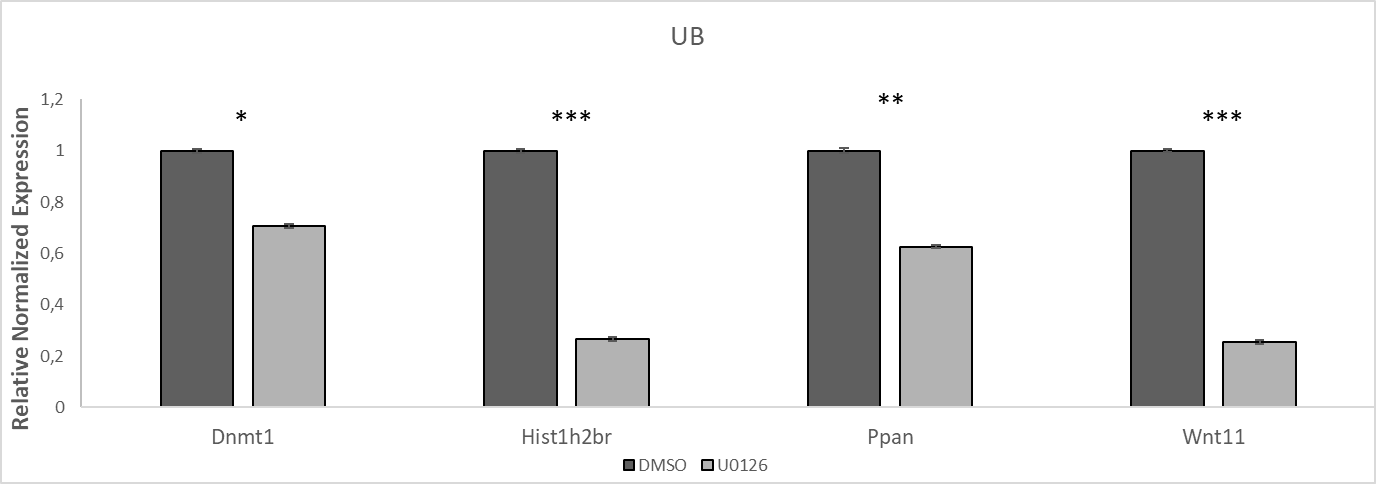


**C**


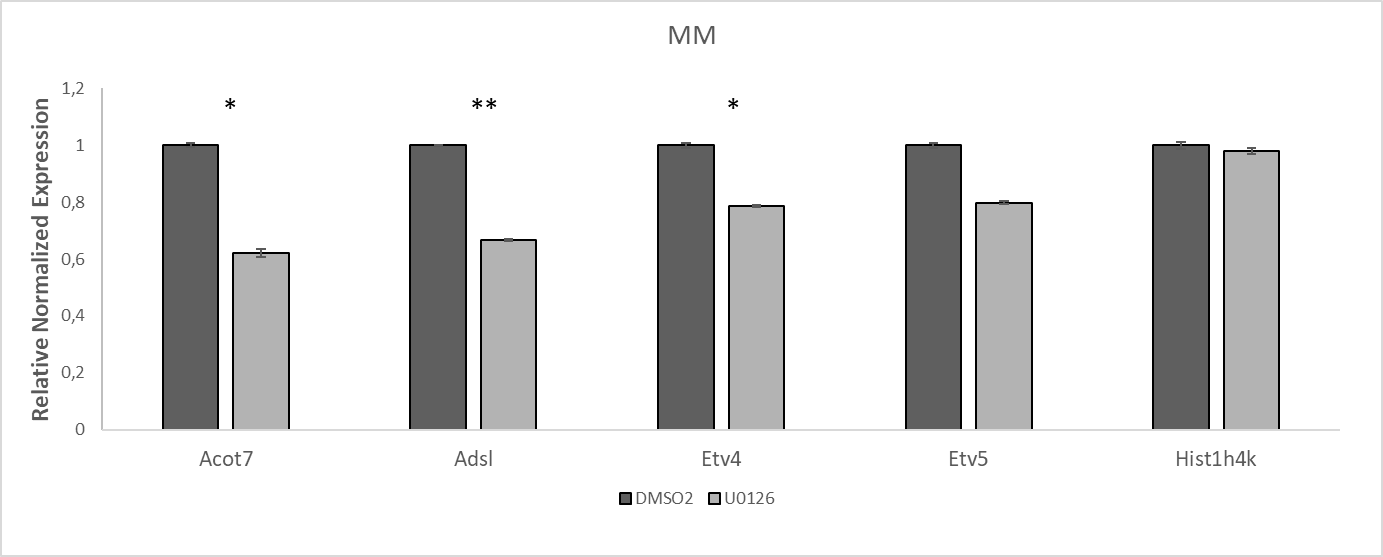


Gene expressions in **(A)** E12.5 whole kidneys (n= 12 kidneys/treatment, three technical replicates), **(B)** isolated ureteric buds (UB, n= 48 kidneys/treatment, three technical, replicates) and **(C)** isolated metanephric mesenchymes (n= 30 kidneys/treatment, three technical replicates) cultured in control (DMSO) or MEK-inhibited (15µM U0126) conditions identifies significant (*=*p<0.05*; **=*p<0.01*; ***=*p<0.001*) changes in a majority of the analyzed transcripts. The red line in **(A)** separates the genes with similar expression changes in UB and/or MM RNA-Seq and whole kidneys (left) from those with opposite expression changes (right).

**Figure S4.** Transcription factor target-enrichment in MAPK/ERK-deficient UB

**
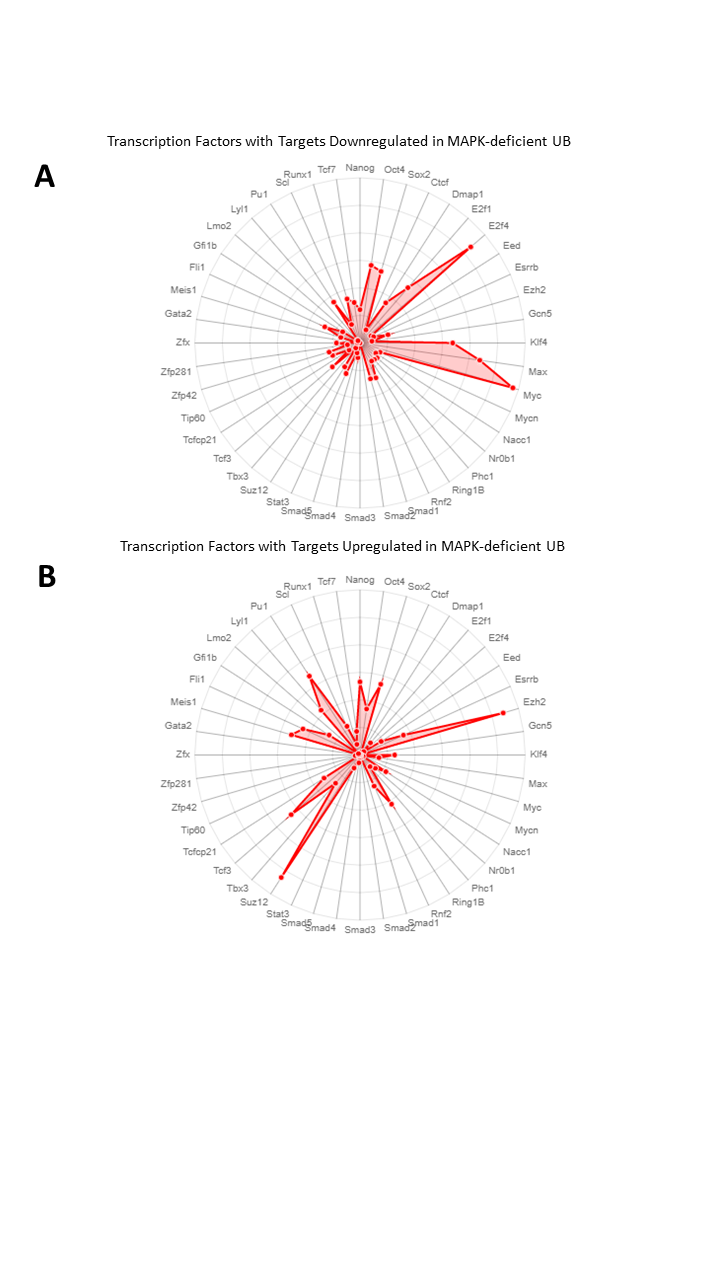
**

Spider charts showing transcription factors with targets downregulated **(A)** and upregulated **(B)** in MAPK-deficient UB (n= 4 kidneys/genotype). StemChecker software was used to compare published transcription factor target genes to those DEGs in our RNA-Seq analysis.


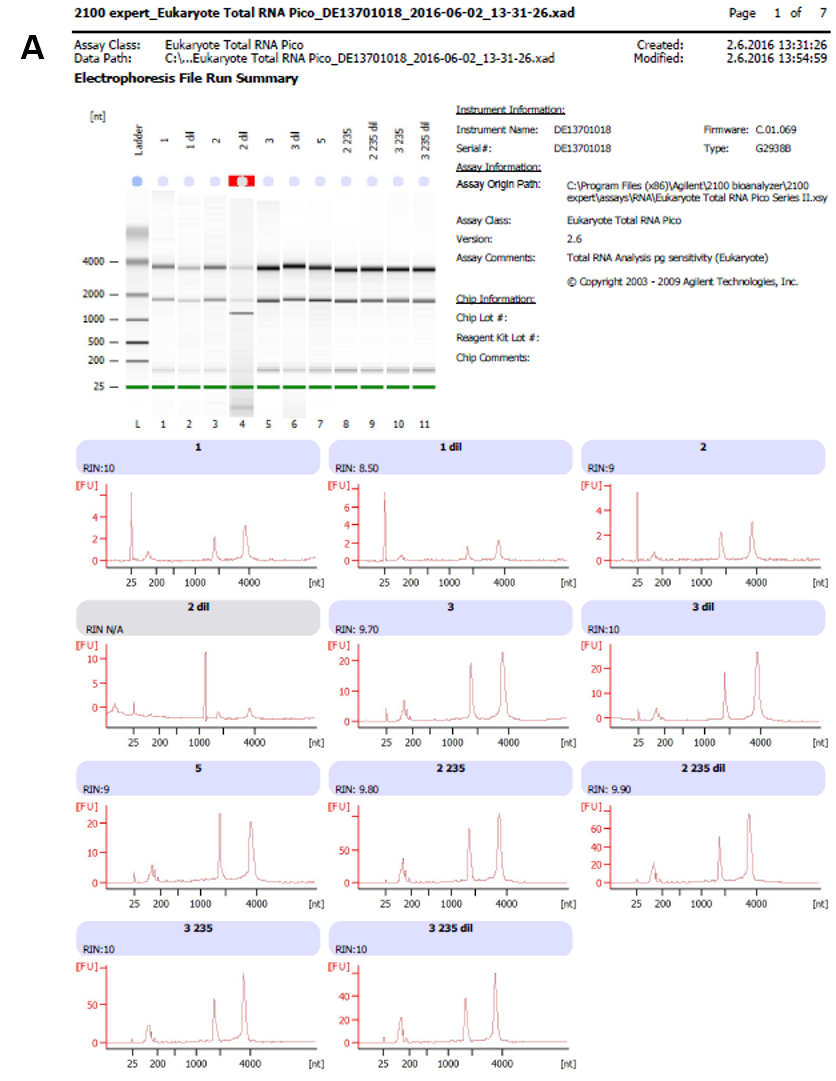
**Figure S5.** Examples of RNA quality assurance studies performed prior proceeding to sequencing of actual samples.

**(A)** An example of the early RNA quality measurements with Bioanalyzer after isolation of control cells from FACS sorted UBs. Different RNAs (1-3, 5, 2 235 and 3 235) (n= 6 kidneys) and their dilutions (dil) are shown.


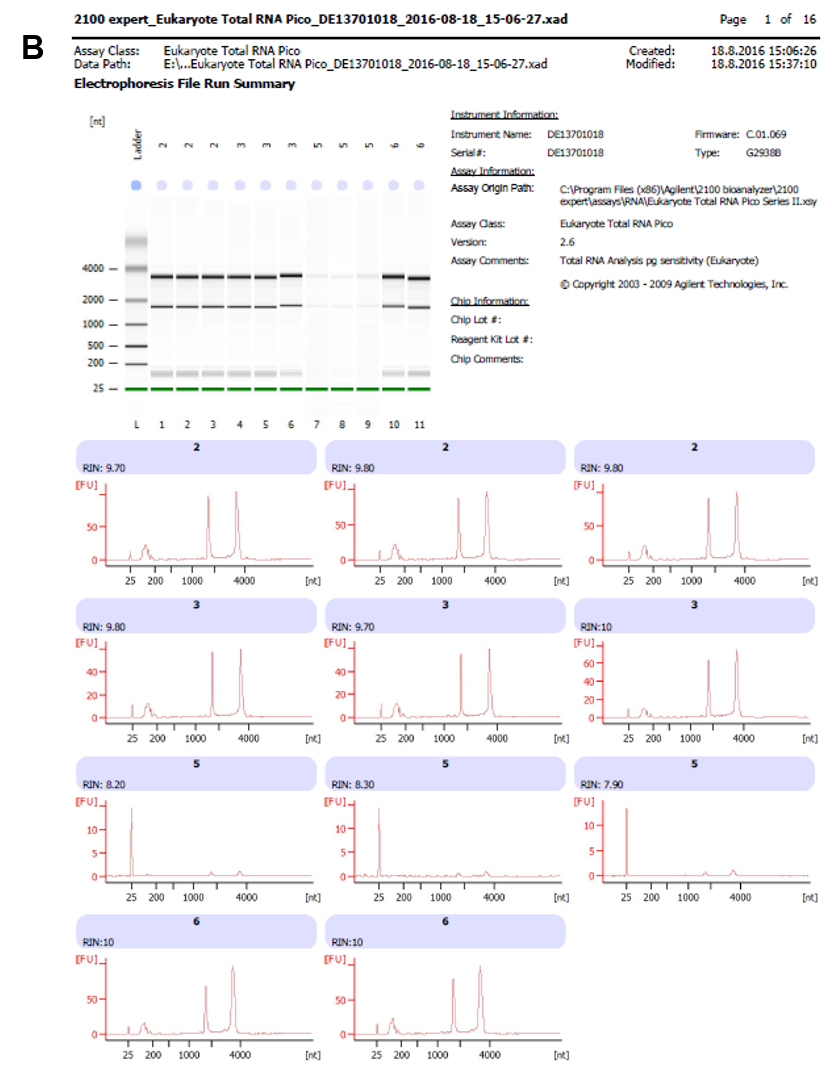


**(B)** An example of the later RNA quality measurements with Bioanalyzer after troubleshooting of RNA isolation protocol of control cells from FACS sorted UBs. Four triplicate control samples (2, 3, 5 and 6, n= 3) were measured and both improved robustness and quality can be seen in comparison to results in A.
